# Vaccine induced mucosal and systemic memory NK/ILCs elicit decreased risk of SIV/SHIV acquisition

**DOI:** 10.3389/fimmu.2024.1441793

**Published:** 2024-09-05

**Authors:** Mohammad Arif Rahman, Isabela Silva de Castro, Luca Schifanella, Massimiliano Bissa, Genoveffa Franchini

**Affiliations:** Animal Models and Retroviral Vaccines Section, Vaccine Branch, Center for Cancer Research, National Cancer Institute, Bethesda, MD, United States

**Keywords:** memory-like NK cells, antigen-reactive ILCs, V2-specific ADCC, innate memory, trained immunity, SIV/SHIV, vaccine (DNA/ALVAC/gp120), cytokines

## Abstract

SIV and HIV-based envelope V1-deleted (ΔV1) vaccines, delivered systemically by the DNA/ALVAC/gp120 platform, decrease the risk of mucosal SIV or SHIV acquisition more effectively than V1-replete vaccines. Here we investigated the induction of mucosal and systemic memory-like NK cells as well as antigen-reactive ILC response by DNA/ALVAC/gp120-based vaccination and their role against SIV/SHIV infection. ΔV1 HIV vaccination elicited a higher level of mucosal TNF-α^+^ and CD107^+^ memory-like NK cells than V1-replete vaccination, suggesting immunogen dependence. Mucosal memory-like NK cells, systemic granzyme B^+^ memory NK cells, and vaccine-induced mucosal envelope antigen-reactive IL-17^+^ NKp44^+^ ILCs, IL-17^+^ ILC3s, and IL-13^+^ ILC2 subsets were linked to a lower risk of virus acquisition. Additionally, mucosal memory-like NK cells and mucosal env-reactive IFN-γ^+^ ILC1s and env- reactive IL-13^+^ ILC2 subsets correlated with viral load control. We further observed a positive correlation between post-vaccination systemic and mucosal memory-like NK cells, suggesting vaccination enhances the presence of these cells in both compartments. Mucosal and systemic memory-like NK cells positively correlated with V2-specific ADCC responses, a reproducible correlate of reduced risk of SIV/HIV infection. In contrast, an increased risk was associated with the level of mucosal PMA/Ionomycin-induced IFN-γ^+^ and CD107^+^ NKG2A^-^NKp44^-^ ILCs. Plasma proteomic analyses demonstrated that suppression of mucosal memory-like NK cells was linked to the level of CCL-19, LT-α, TNFSF-12, and IL-15, suppression of systemic env-reactive granzyme B^+^ memory-like NK cells was associated with the level of OLR1, CCL-3, and OSM, and suppression of IL-17^+^ ILCs immunity was correlated with the level of IL-6 and CXCL-9. In contrast, FLT3 ligand was associated with promotion of protective mucosal env-reactive IL-17^+^ responses. These findings emphasize the importance of mucosal memory-like NK cell and envelope- reactive ILC responses for protection against mucosal SIV/SHIV acquisition.

## Introduction

An effective, distributable vaccine is needed to control the global HIV epidemic, which persists despite major advances in pre-exposure and post-exposure treatment. Though significant, the accessibility of treatment is inadequate, as more than 39 million people are now living with HIV, with 1.3 million new infections estimated in 2022 alone (1). One of the key elements of vaccine efficacy in human and non-human primate HIV/SIV trials is the induction of trained immunity, or adaptive “innate memory” responses (2–4). The innate immune system, which includes natural killer (NK) cells and innate lymphoid cells (ILCs), provides the initial host responses upon encountering pathogens. Though NK cells and ILCs have conventionally been considered part of the innate immune system, recent reports suggest these cells also have memory properties (5–20). Adaptive “memory-like” cells have been detected in mice, macaques, and humans (5–14, 20), and could potentially contribute to more potent vaccine efficacy. Three types of memory-like NK cells have been described: (1) hapten and viral antigen-reactive memory-like NK cells with long-lived memory; (2) NKG2C^+^ and Ly49H^+^ memory-like NK cells in cytomegalovirus (CMV) infected humans and mice, respectively; (3) cytokine-stimulated memory-like NK cells that produce enhanced IFN-γ after re-stimulation (21). One of the mechanisms by which NK cells eliminate virus-infected cells is antibody-dependent cellular cytotoxicity (ADCC), which has been associated with protective HIV/SIV vaccine outcomes in humans (22) and in macaques (23–26). NK cells become activated when their FcgRIIIa (CD16a) receptors interact with the Fc region of IgG antibodies attached to viral antigens on the surface of infected cells, which triggers the release of perforin, granzyme, and various cytokines, promoting ADCC and facilitating the elimination of virally infected cells. Memory-like NK cells have been shown to induce ADCC activity (27). Nonetheless, the correlation between memory-like NK cells and ADCC responses, as well as their involvement in SIV/HIV infection, remains incompletely understood.

The study of mucosal NK/ILCs in humans is particularly challenging due to the difficulty of obtaining mucosal biopsies for longitudinal study and the fact that ILCs are more abundant in tissue and less available in peripheral blood (28). However, the macaque model is a reliable parallel of HIV infection in humans (29) and provides a viable model to investigate mucosal NK/ILCs. ILCs can be divided into three types depending on their transcription factor expression. Type 1 ILCs (ILC1s) express T-bet transcription factor and produce IFN-γ (30). Type 3 ILCs (ILC3s) express RORγt and secrete IL-17 and IL-22, sharing similarities with TH17 and TH22 cells (31). Non-ILC1 and non-ILC3 cells are defined here as type 2 ILCs (ILC2s), which are the Th2 counterpart of the innate immune system and secrete type 2 cytokines such as IL-4, IL-5, and IL-13 (32). Since ILCs have plasticity and can change their phenotype and functionality, the classification of these cells is not absolute (30, 33). Recent studies suggest memory properties in all ILC types; ILC1, ILC2, and ILC3 cells all showed recall responses in the murine model, in murine cytomegalovirus (MCMV)-infected, hapten/allergen sensitized mice, and in the enterobacterial mouse model (15–19). NKp44^+^ ILCs are restricted to the mucosa of rhesus macaques and closely resemble human mucosal NK22 cells (34). The env-reactive mucosal NKp44^+^ ILC response in DNA/ALVAC/gp120-vaccinated monkeys and its protective role in SIV/SHIV infection has been well described (11, 23, 26, 35). However, the role of SIV/HIV vaccine-induced mucosal memory-like NK cells and antigen-reactive ILC1, ILC2, and ILC3 responses, and their potential contribution to protection from infection, has been inadequately explored.

We have previously demonstrated that post-vaccination mucosal env-reactive IL-17^+^ NKp44^+^ cells as well as PMA/Ionomycin-induced IFN-γ^+^ NKG2A^-^NKp44^-^ ILCs are associated with reduced or increased risk of SIV/SHIV acquisition, respectively (11, 23, 35). However, longitudinal studies of mucosal NK/ILCs were not previously conducted to understand the effect of vaccination on these cells. We met this need by performing longitudinal studies in the macaque model, in which we evaluated different mucosal NK/ILCs subsets to understand their antigen-reactivity. First, we focused on mucosal memory-like NK cells, which were characterized as NKG2A^+^ cells lacking expression of spleen tyrosine kinase (syk) and Fc*ϵ*R1*γ*, since epigenetic analysis showed syk and Fc*ϵ*R1*γ* down-regulation in memory-like NK cells (14, 36). We further analyzed the three ILC types, classified based on their transcription factor expression in the system described by Bal, et al. (37). Next, we focused on NK/ILCs based on their expression of NKG2A and NKp44 markers, which have previously been studied with regard to SIV/SHIV vaccination and subsequent infection (11, 23, 35). Lastly, we evaluate all these NK/ILC cell types in the blood to understand their reactivity to env-antigen as well as their role in protection from SIV infection. We observed that DNA/ALVAC-based vaccination with SIV or HIV antigens induced mucosal/systemic memory-like NK cells as well as mucosal antigen-reactive ILCs. Post-vaccination plasma cytokine/chemokine levels were associated with either promotion or suppression of memory-like NK/ILCs. To our knowledge, this is the first report showing that DNA/ALVAC-based vaccination can induce trained immunity by generating mucosal adaptive “memory-like” NK cells as well as antigen-reactive ILC responses. Because these memory NK/ILCs correlated with protective ADCC responses and a reduced risk of SIV/SHIV acquisition as well as with control of post-infection viral load (VL), we conclude they might be essential for protection against SIV/HIV infection.

## Results

### DNA/ALVAC/gp120 vaccine induces mucosal memory-like antigen-reactive NK cells against SIV/SHIV

**Figure 1:**
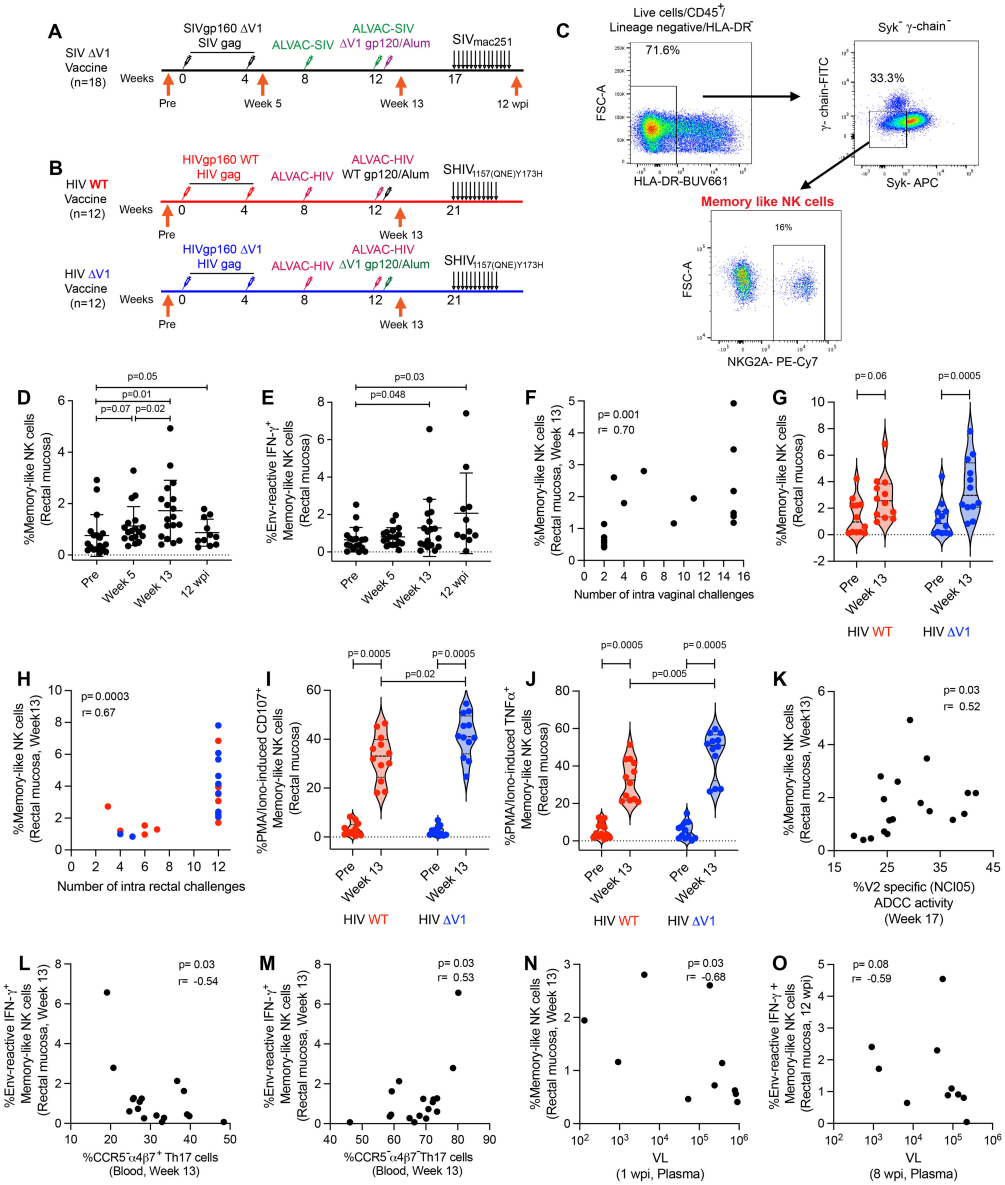
Immunization regimen and identification of memory-like NK cell responses in mucosa. **(A)** A group of 18 female, rhesus macaques (ΔV1 SIV vaccine) was primed with DNA-SIVgp160 ΔV1+SIV_mac239_ *gag* and boosted with ALVAC-SIV encoding *env*, *gag*, and *pol* and ALVAC-SIV+ΔV1 gp120 protein in alum hydroxide at the indicated timepoints. Beginning at week 17, protective efficacy against SIV_mac251_ was assessed by subjecting all animals to up to 14 weekly intravaginal viral exposures (arrows) until infection was confirmed. The animals were challenged intravaginally weekly with 1 ml of a SIV_mac251_ stock (23, 24, 69, 70) containing 800 median tissue culture infectious doses (TCID50). **(B)** A subset of 12 male rhesus macaques (WT HIV vaccine) was primed with DNA-HIVgp160+HIV *gag* and boosted with ALVAC-HIV encoding *env*, *gag*, and *pol* and ALVAC-HIV+gp120 protein in alum hydroxide at the indicated timepoints. Another subset of 12 male rhesus macaques (ΔV1 HIV vaccine) was primed with DNA-HIVgp160 ΔV1+HIV *gag* and boosted with ALVAC-HIV encoding *env*, *gag*, and *pol* and ALVAC-HIV+ΔV1 gp120 protein in alum hydroxide at the indicated timepoints. Beginning at week 21, protective efficacy against SHIV was assessed by subjecting all animals to up to 11 weekly intrarectal viral exposures (arrows) until infection was confirmed. The animals were challenged intrarectally weekly with 1 ml of a SHIV_157(QNE)Y173H_ (26, 64) diluted 1:10,000 from stock. **(C)** Gating strategy of memory-like NK cells. **(D, E)** Evaluation of **(D)** memory-like NK cells and **(E)** env-reactive IFN-γ^+^ memory-like NK cells over the course of vaccination in the female macaques. **(F)** Correlation of memory-like NK cells at week 13 in the female macaques with number of challenges. **(G)** Evaluation of memory-like NK cells over the course of vaccination in the subsets of male macaques. **(H)** Correlation of memory-like NK cells at week 13 in all 24 male macaques with number of SHIV challenges. **(I, J)** Evaluation of **(I)** PMA/Ionomycin -induced CD107^+^ memory-like NK cells and **(J)** PMA/Ionomycin -induced TNF-α^+^ memory-like NK cells over the course of vaccination in the subsets of male macaques. **(K)** Correlation of memory-like NK cells with V2-specific ADCC. **(L, M)** Correlation of env-reactive IFN-γ^+^ memory-like NK cells with **(L)** CCR5^-^α4β7^+^Th17 cells and **(M)** CCR5^-^α4β7^-^Th17 cells in blood. **(N)** Correlation of memory-like NK cells with VL at 1 wpi. **(O)** Correlation of env-reactive IFN-γ^+^ memory-like NK cells with VL at 8 wpi. Data shown in **(C, D, G, I, J)** were analyzed with Wilcoxon signed-rank test or Mann-Whitney test. Data shown in **(E, H, K-O)** were analyzed by the Spearman correlation test. Horizontal and vertical bars denote mean and SD, respectively. Violin plot vertical bars denote median and quartiles. Here, black, red and blue symbols represent ΔV1 SIV vaccinated female macaques, WT HIV vaccinated male macaques and ΔV1 HIV vaccinated male macaques, respectively.

Here we report two animal studies designed to investigate mucosal antigen-reactive NK/ILC responses in female animals vaccinated with ΔV1 SIV, or male animals immunized with wild type (WT) HIV or ΔV1 HIV env immunogens. In the first study (ΔV1 SIV), we vaccinated 18 female macaques as described in the methods and previously (23). To understand the effect of vaccination on different subsets of mucosal NK/ILCs, we collected mucosal tissue from animal rectal biopsies before vaccination (pre), 1 week post 2nd prime (week 5), 1 week post 2nd boost (week 13) and 12 weeks post infection (12 wpi; **Figure 1A**). In the second study, we vaccinated a total of 24 male macaques, 12 in the WT HIV group and 12 in the ΔV1 HIV group, as described in the methods (**Figure 1B**). We investigated the effect of vaccination on mucosal memory-like NK cells, which we defined as CD45^+^ lineage^-^HLA-DR^-^ Syk^-^ γ-chain(Fc*ϵ*R1*γ*)^-^ NKG2A^+^ cells (10, 11), since all NK cells in rhesus macaques express NKG2A (38) (**Figure 1C**). In the first study, the frequency of memory-like NK cells showed a trend of increasing after priming, increasing significantly after boost and remaining elevated at 12 wpi (**Figure 1D**). The frequency of memory-like NK cells at 12 wpi was comparable to the frequency at one week post vaccination (week 13) (p=0.41), suggesting there was no further expansion post-infection of the cells that had already expanded due to vaccination. We next investigated whether these memory-like NK cells showed responses against SIV/SHIV envelope antigen. At post boost and 12 wpi, memory-like NK cells showed IFN-γ responses upon stimulation with overlapping SIV gp120 peptides compared to baseline (**Figure 1E**), suggesting the vaccine-induced memory-like NK cells were antigen-reactive. IFN-γ^+^ memory-like NK cells induced by PMA/Ionomycin stimulation showed their highest responses at week 13 and remained elevated 12 wpi compared to baseline (**Supplementary Figure S1A**). Interestingly, the frequency of memory-like NK cells at week 13 positively correlated with number of challenges (**Figure 1F**), however, we did not observe any protective correlation with overall NKG2A^+^ NK cells (p=0.44, r=0.1) or non-memory NK cells (p=0.65, r=0.1) (data not shown), suggesting a protective role for memory-like NK cells against SIV challenges.

While only a trend of increased memory-like NK cell frequency was observed for WT HIV vaccination, a significant increase was observed for ΔV1 HIV vaccination (**Figure 1G**). The frequency of memory-like NK cells did not differ between the two groups at week 13, and only 7 of the combined 24 animals became infected among the two group of animals. Thus, we combined the subgroups for correlation analysis. As observed in the ΔV1 SIV vaccine (**Figure 1F**), the frequency of memory-like NK cells also positively correlated with the number of challenges in the HIV vaccinated macaques (**Figure 1H**). In this HIV vaccine study, we did not stimulate the cells with HIV-specific antigens. However, the ΔV1 HIV vaccine showed higher CD107 (**Figure 1I**) and TNF-α responses (**Figure 1J**) compared to WT HIV vaccine upon PMA/Ionomycin stimulation, suggesting that the functionality of memory-like NK cell differed from one vaccine to the other. Interestingly, mucosal NK cells showed comparable levels of CD107 (**Supplementary Figure S1B**) and TNF-α responses (**Supplementary Figure S1C**) in the two groups of animals. Furthermore, IFN-γ responses by memory-like NK cells were comparable between the two vaccine groups (**Supplementary Figure S1D**).

In previous studies using the DNA/ALVAC/gp120 platform, V2-specific ADCC was established as one of the correlates of protection of the DNA/ALVAC/gp120ΔV1 vaccines in the macaque model (23–25). Thus, we investigated whether memory-like NK cells affect V2-specific ADCC. Minimal ADCC killing activity against gp120-coated target cells in the naïve animals was observed (**Supplementary Figure S1E**), thus, V2-specific ADCC activity in the baseline was not measured. We observed a positive correlation between memory-like mucosal NK cells and V2-specific ADCC for the SIV ΔV1 vaccine (**Figure 1K**) and a positive trend for the WT and ΔV1 HIV vaccines (**Supplementary Figure S1F**), suggesting memory-like NK cells might promote V2-specific ADCC and thereby contribute to protective efficacy.

We further evaluated the role of memory-like NK cells and cytokine producing NK cells in influencing the frequency of T cells in the ΔV1 SIV vaccinated female macaques. We observed a negative correlation of env-reactive IFN-γ^+^ memory-like NK cells with CCR5^-^α4β7^+^ Th17 cells (**Figure 1L**) and a positive correlation with CCR5^-^α4β7^-^ Th17 cells (**Figure 1M**). The role of these T cells in HIV/SIV control is not well known, however, these data suggested that memory-like NK cells might influence the T cell responses. Moreover, a negative correlation between mucosal memory-like NK cells at week 13 and VL at 1 wpi (**Figure 1N**), as well as env- reactive IFN-γ^+^ memory-like NK cells at 12 wpi and VL at 8 wpi was observed (**Figure 1O**), suggesting memory-like NK cells play a role in the control of VL, and VL also influences the frequency of env-reactive IFN-γ^+^ memory-like NK cells.

### Mucosal antigen-reactive ILCs induced by HIV vaccine candidates

**Figure 2:**
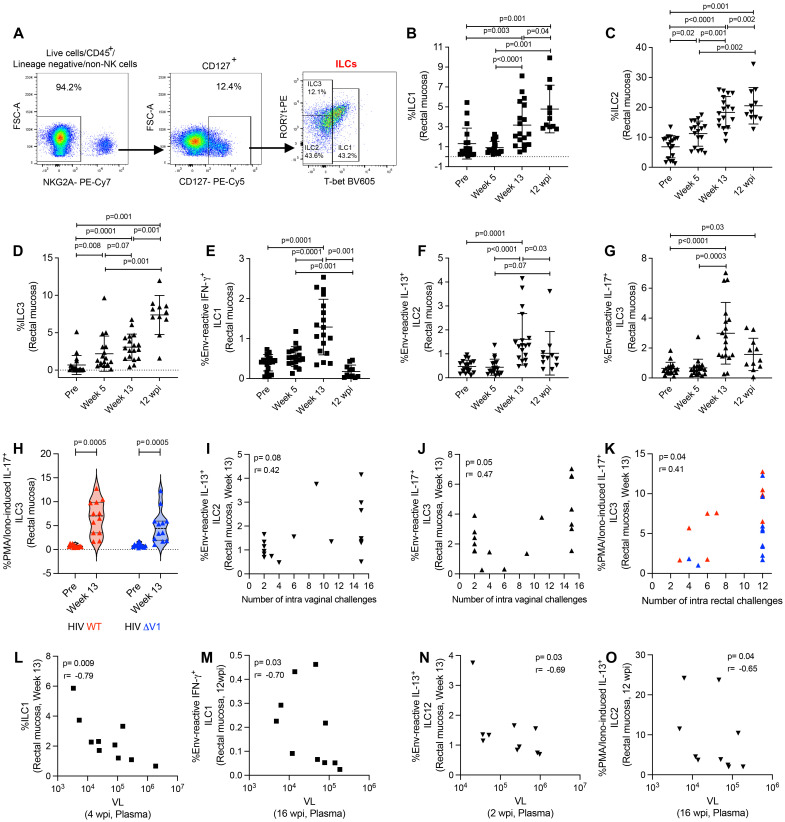
Evaluation of antigen-reactive ILC responses in mucosa. **(A)** Gating strategy of ILCs. **(B-H)** Evaluation of **(B)** ILC1, **(C)** ILC2, **(D)** ILC3, **(E)** env-reactive IFN-γ^+^ ILC1, **(F)** env-reactive IL-13^+^ ILC2, **(G)** env-reactive IL-17^+^ ILC3, over the course of vaccination of female macaques and **(H)** PMA/Ionomycin -induced IL-17^+^ ILC3 over the course of vaccination of male macaques. **(I-K)** Correlation of **(I)** env-reactive IL-13^+^ ILC2, **(J)** env-reactive IL-17^+^ ILC3, and **(K)** PMA/Ionomycin -induced IL-17^+^ ILC3 with number of challenges. **(L-O)** Correlation of **(L)** ILC1, **(M)** env-reactive IFN-g^+^ ILC1, **(N)** env-reactive IL-13^+^ ILC2, and **(O)** PMA/Ionomycin -induced IL-13^+^ ILC2 with VL. Data shown in **(B-H)** were analyzed with Wilcoxon signed-rank test or Mann-Whitney test. Data shown in **(I-O)** were analyzed by the Spearman correlation test. Horizontal and vertical bars denote mean and SD. Violin plot vertical bars denote median and quartiles. Here, black, red and blue symbols represent ΔV1 SIV vaccinated female macaques, WT HIV vaccinated male macaques and ΔV1 HIV vaccinated male macaques, respectively.

Here, we classified type 1, 2, and 3 ILCs based on the expression of transcription factors (30, 31, 37). We used CD45^+^lineage^-^NKG2A^-^ cells for ILC gating, and defined ILC1 as T-bet^+^ cells, ILC2 as T-bet^-^RORγt^-^ cells, and ILC3 as T-bet^-^RORγt^+^ cells (**Figure 2A**). We observed increased frequencies of ILC1 (**Figure 2B**), ILC2 (**Figure 2C**), and ILC3 (**Figure 2D**) cells over the course of ΔV1 SIV vaccination and post WT HIV and ΔV1 HIV vaccination (**Supplementary Figures S2A-C**). The frequency of these cells in the ΔV1 SIV vaccinated macaques further increased 12 wpi (**Figures 2B-D**). Interestingly, the ILC1, ILC2, and ILC3 cell populations showed antigen reactivity when stimulated with overlapping gp120 peptides. One week post last immunization (week 13), the ΔV1 SIV vaccinated animals showed an increased frequency of env-reactive IFN-γ^+^ ILC1 (**Figure 2E**), env-reactive IL-13^+^ ILC2 (**Figure 2F**), and env-reactive IL-17^+^ ILC3 (**Figure 2G**). Env-reactive IL-17^+^ ILC3 (**Figure 2G**) remained elevated compared to baseline even after 12 wpi. We also observed elevated PMA/Ionomycin-induced responses at week 13 for all subtypes of ILCs in ΔV1 SIV-vaccinated animals (**Supplementary Figures S2D-F**), as well as in HIV-vaccinated animals (**Figure 2H**; **Supplementary Figures S2G, H**). Env- reactive IL-13^+^ ILC2 showed a trend of positive correlation (**Figure 2I**) while IL-17^+^ ILC3 (**Figure 2J**) positively correlated with number of challenges in SIV ΔV1-vaccinated animals. Furthermore, PMA/Ionomycin-induced IL-17^+^ ILC3 (**Figure 2K**) positively correlated with the number of challenges in all HIV vaccinated animals. Moreover, in the ΔV1 SIV vaccinated female infected macaques, a negative correlation was observed between ILC1 at week 13 and VL at 4 wpi (**Figure 2L**), env-reactive IFN-γ^+^ILC1 at 12 wpi and VL at 16 wpi (**Figure 2M**), env- reactive IL-13^+^ ILC2 at week 13 and VL at 2 wpi (**Figure 2N**), PMA/Ionomycin-induced IL-13^+^ ILC2 at 12 wpi and VL at 16wpi (**Figure 2O**), PMA/Ionomycin-induced IL-13^+^ ILC2 at 12 wpi and VL at 12 wpi (**Supplementary Figure S2I**), PMA/Ionomycin-induced IL-13^+^ ILC2 at week 13 and VL at 1 wpi (**Supplementary Figure S2J**), PMA/Ionomycin-induced IFN-γ^+^ ILC1 at week 13 and VL at 2wpi (**Supplementary Figure S2K**), suggesting the role of these ILCs in VL control. We further observed a negative correlation between ILC1 at 12 wpi and VL at 1 wpi (**Supplementary Figure S2L**), env reactive IL-17^+^ ILC3 at 12 wpi and VL at 4 wpi (**Supplementary Figure S2M**), env reactive IL-17^+^ ILC3 at 12 wpi and VL at 6 wpi (**Supplementary Figure S2N**), suggesting VL also influence the frequency of these ILCs. Taken together, these data suggested that DNA/ALVAC/gp120-based vaccination was able to generate antigen-reactive ILC responses, and these responses were associated with reduced risk of SIV infection and VL control.

We further evaluated the role of ILCs on T cell responses in the ΔV1 SIV vaccinated female infected macaques. In the SIV macaque model, CCR5^-^α4β7^+^ CD4^+^ T cells have been associated with decreased acquisition risk, and, in contrast, CCR5^+^α4β7^+^ CD4^+^ T cells are correlated with increased risk of SIV acquisition (4). Here, vaccination decreased the frequency of CCR5^-^α4β7^+^ Th1 cells (**Supplementary Figure S3A**) and CCR5^-^α4β7^+^ Th2 cells (**Supplementary Figure S3B**). Env-reactive IL-13^+^ ILC2 at week 13 positively correlated with vaccine induced CCR5^-^α4β7^+^ Th1 cells (**Supplementary Figure S3C**). PMA/Ionomycin-induced and env-reactive IL-17^+^ ILC3 at week 13 positively correlated with vaccine induced CCR5^-^α4β7^+^ Th2 cells (**Supplementary Figure S3D**) and with CCR5^-^α4β7^+^ Th2 cells at week 13 (**Supplementary Figure S3E**), respectively. Furthermore, vaccination decreased the frequency of CCR5^+^α4β7^+^ Th1 cells (**Supplementary Figure S3F**) and these cells negatively correlated with env-reactive IL-17^+^ ILC3 (**Supplementary Figure S3G**). Taken together, these data suggested that ILCs promote the protective T cells, whereas, *ILCs* were negatively associated with non-protective T cells.

### Vaccination induces mucosal NK cells, not antigen-reactive NK responses

**Figure 3:**
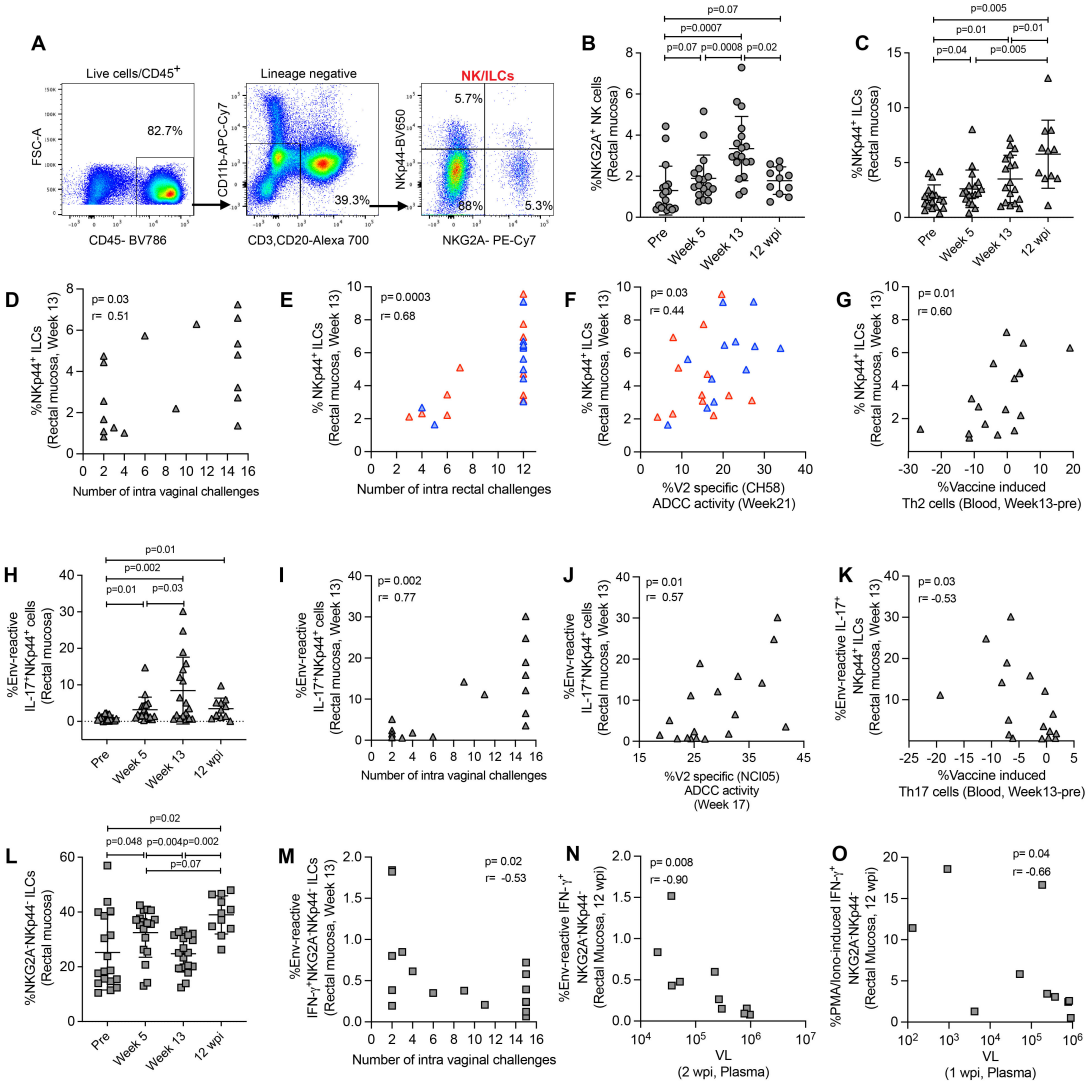
Evaluation of NK/ILC responses in mucosa. **(A)** Gating strategy of NK/ILCs. **(B, C)** Evaluation of **(B)** NKG2A^+^ NK cells and **(C)** NKp44^+^ ILCs over the course of vaccination of female macaques. **(D, E)** Correlation of NKp44^+^ ILCs with number of challenges in the **(D)** female and **(E)** male macaques. **(F, G)** Correlation of NKp44^+^ ILCs with **(F)** V2-specific ADCC and **(G)** vaccine induced Th2 cells. **(H)** Evaluation of env-reactive IL-17^+^ NKp44^+^ ILCs over the course of vaccination of female macaques. **(I-K)** Correlation of env-reactive IL-17^+^ NKp44^+^ ILCs with **(H)** number of challenges, **(I)** V2-specific ADCC and **(K)** vaccine induced Th17 cells in the female macaques. **(L)** Evaluation of NKG2A^-^ NKp44^-^ ILCs over the course of vaccination of female macaques. **(M, N)** Correlation of env-reactive IFN-γ^+^ NKG2A^-^ NKp44^-^ ILCs with **(M)** number of challenges and **(N)** VL in female macaques. **(O)** Correlation of PMA/Ionomycin -induced IFN-γ^+^ NKG2A^-^ NKp44^-^ ILCs with VL in female macaques. Data shown in **(B, C, H, L)** were analyzed with Wilcoxon signed-rank test. Data shown in **(D-G, I-K, M-O)** were analyzed by the Spearman correlation test. Horizontal and vertical bars denote mean and SD, respectively. Here, gray, red and blue symbols represent ΔV1 SIV vaccinated female macaques, WT HIV vaccinated male macaques and ΔV1 HIV vaccinated male macaques, respectively.

NKG2A has been identified as a NK cell marker in rhesus macaques (38). In this study, we defined NK cells, NKp44^+^ ILCs, and NKG2A^-^NKp44^-^ ILCs by the expression of NKG2A and the NKp44 marker (11, 23, 35). We used CD45^+^lineage^-^ cells for NK/ILC gating. NK cells were gated as NKG2A^+^ cells, NKp44 ILCs were gated as NKp44^+^ cells, and NKG2A^-^NKp44^-^ double negative ILCs were gated as NKG2A^-^NKp44^-^ cells (**Figure 3A**). We observed an increase in mucosal NK cells after vaccination in ΔV1 SIV vaccinated macaques (**Figure 3B**) and a similar trend in HIV vaccinated animals (**Supplementary Figure S4A**). In ΔV1 SIV vaccinated macaques, the frequency of NK cells showed a trend to decrease to baseline levels at 12 wpi (**Figure 3B**). We did not observe any increase of antigen-reactive immune responses for IFN-γ^+^ NKG2A^+^ cells (**Supplementary Figure S4B**), however, upon PMA/Ionomycin stimulation a significant increase of IFN-γ^+^ NKG2A^+^ cells was observed at week 13 for both ΔV1 SIV and HIV groups (**Supplementary Figures S4C, D**), which returned to baseline by 12 wpi in the ΔV1 SIV group (**Supplementary Figure S4C**). These cells were not associated with protective response against SIV/SHIV acquisition or with VL (data not shown), suggesting mucosal total NK cells were not antigen-reactive and did not provide protection against SIV/SHIV infection.

### Mucosal NKp44^+^ ILCs induced by vaccination are linked to a decreased risk of both vaginal SIVmac251 and rectal SHIV infection

The frequency of mucosal NKp44^+^ ILCs increased after vaccination in both SIV ΔV1 vaccinated animals (**Figure 3C**) and HIV vaccinated animals (**Supplementary Figure S4E**). The frequency of NKp44^+^ cells significantly increased in ΔV1 SIV vaccinated macaques at 12 wpi compared to all timepoints (**Figure 3C**). At week 13, NKp44^+^ ILC frequency was associated with reduced risk of SIV/SHIV infection (**Figures 3D, E**). Interestingly, the frequency of NKp44^+^ ILCs in HIV vaccinated animals was positively associated with V2-specific ADCC activity (**Figure 3F**) and in ΔV1 SIV vaccinated macaques negatively associated with vaccine induced Th2 cells (**Figure 3G**) and with vaccine induced Th17 cells (**Supplementary Figure S4F**). Moreover, the frequency of env antigen-reactive IL-17^+^ NKp44^+^ cells increased significantly upon ΔV1 SIV vaccination and remained elevated even after 12 wpi (**Figure 3H**). At week 13, their frequency was associated with reduced risk of SIV infection (**Figure 3I**), positively correlated with V2-specific ADCC activity animals (**Figure 3J**) and negatively correlated with vaccine induced Th17 cells in the ΔV1 SIV vaccinated animals (**Figure 3K**). At week 13, the frequency of PMA/Ionomycin induced IL-17^+^ NKp44^+^ cells significantly increased in SIV ΔV1 (**Supplementary Figure S4G**) and HIV vaccinated animals (**Supplementary Figure S4H**) and was associated with reduced risk of SIV/SHIV infection (**Supplementary Figures S4I, J**). PMA/Ionomycin induced IL-17^+^ NKp44^+^ cells at week 13 were positively correlated with V2-specific ADCC activity (**Supplementary Figure S4K**) and vaccine induced CCR5^+^α4β7^+^ Th1 cells in SIV ΔV1 vaccinated animals (**Supplementary Figure S4L**). Together, these results show that vaccination induces env-reactive NKp44^+^ ILCs, which promote ADCC activity, influence T cells and enhance protection from infection.

### Rectal NKG2A^-^NKp44^-^ ILCs expressing IFN-γ^+^ associated with increased risk of both vaginal SIV_mac251_ and rectal SHIV infection

At week 13, the frequency of NKG2A^-^NKp44^-^ double negative ILCs was comparable to baseline in ΔV1 SIV vaccinated animals (**Figure 3L**) and HIV vaccinated animals (**Supplementary Figure S5A**), but significantly increased at 12 wpi compared to pre and week 13 timepoints in ΔV1 SIV macaques (**Figure 3L**). We did not observe an increase in env-reactive IFN-γ^+^ NKG2A^-^NKp44^-^ double negative ILCs over the course of this study (**Supplementary Figure S5B**), however, at week 13 these cells were negatively associated with the number of SIV challenges (**Figure 3M**). Upon PMA/Ionomycin stimulation, IFN-γ^+^ (**Supplementary Figures S5C, D**) and CD107a^+^ (**Supplementary Figure S5E**) NKG2A^-^NKp44^-^ double negative ILCs increased post vaccination in all vaccinated macaques and IFN-γ^+^ NKG2A^-^NKp44^-^ ILCs returned to baseline at 12 wpi in the ΔV1 SIV group (**Supplementary Figure S5C**). Env-reactive (**Figure 3N**) and PMA/Ionomycin-induced (**Figure 3O**) IFN-γ^+^ NKG2A^-^NKp44^-^ double negative ILCs at 12 wpi negatively correlated with VL at 2 wpi and at 1 wpi, respectively, suggesting viremia decreases these mucosal cells at a later phase of infection. Moreover, env-reactive IFN-γ^+^ NKG2A^-^NKp44^-^ double negative ILCs negatively correlated with protective CCR5^-^α4β7^+^ Th2 cells (**Supplementary Figure S5F**) and positively correlated with protective CCR5^-^α4β7^-^ Th2 cells (**Supplementary Figure S5G**). Furthermore, PMA/Ionomycin-induced IFN-γ^+^ NKG2A^-^NKp44^-^ double negative ILCs positively correlated with vaccine induced Th2 cells (**Supplementary Figure S5H**), suggesting the interplay of IFN-γ^+^ NKG2A^-^NKp44^-^ cells and T cells. PMA/Ionomycin induced cytokine producing double negative ILCs negatively correlated with the number of challenges in both the female and male macaques (**Supplementary Figures S5I-K**), indicating that NKG2A^-^NKp44^-^ double negative ILCs play a role in increased risk of SIV/SHIV acquisition.

### Systemic memory NK cells, not systemic antigen-reactive ILCs play a protective role against SIV infection

**Figure 4:**
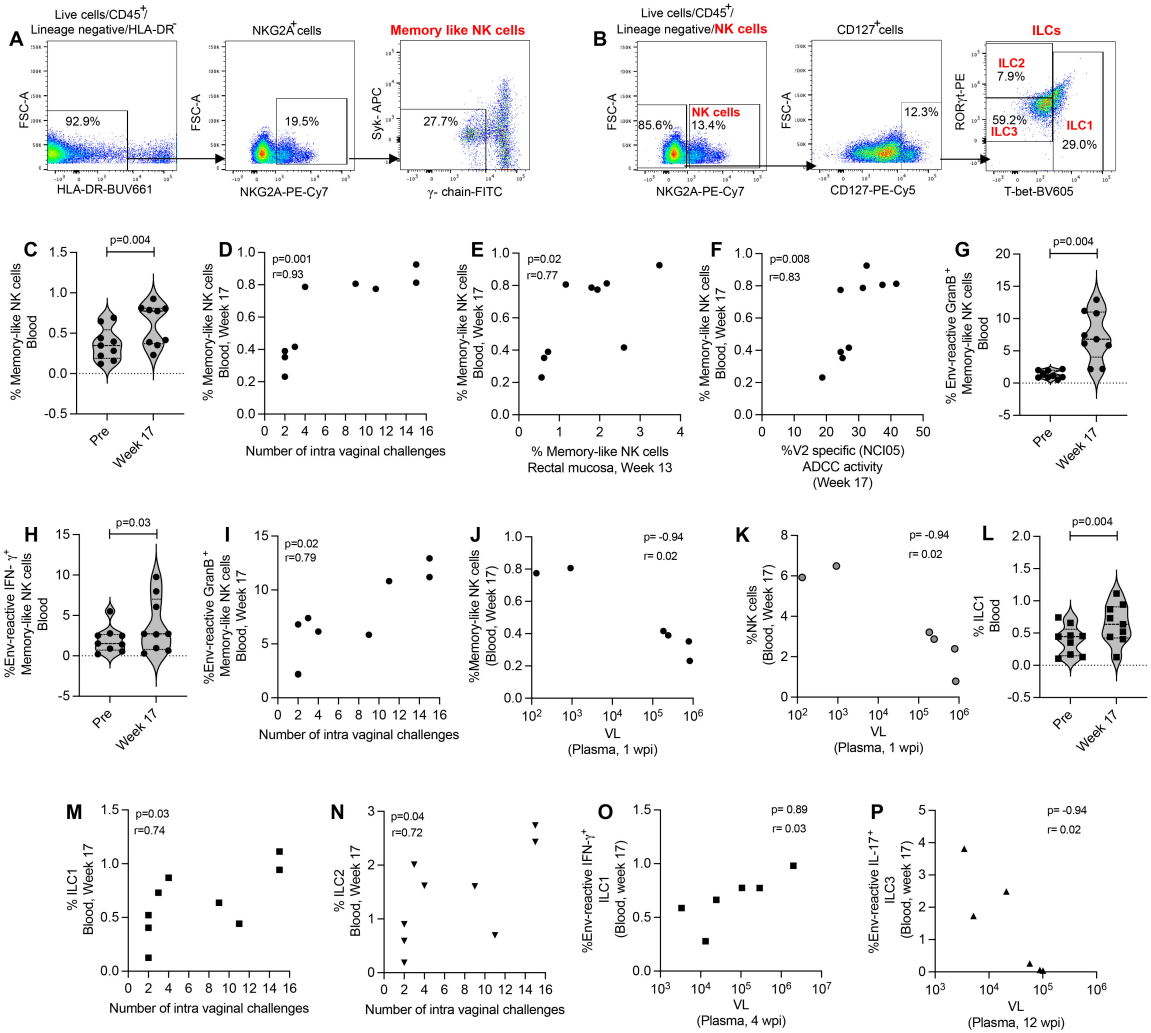
Evaluation of systemic NK/ILC responses in female macaques. **(A, B)** Gating strategy of NK/ILCs. **(C)** Evaluation of memory-like NK cells over the course of vaccination of female macaques. **(D)** Correlation of systemic memory-like NK cells with number of challenges. **(E)** Correlation of systemic and mucosal memory-like NK cells. **(F)** Correlation of systemic memory-like NK cells with V2-specific ADCC. **(G, H)** Evaluation of **(G)** env-reactive GranB^+^ memory-like NK cells and **(H)** env-reactive IFN-γ^+^ memory-like NK cells over the course of vaccination of female macaques. **(I)** Correlation of systemic env-reactive GranB^+^ memory-like NK cells with number of challenges. **(J, K)** Correlation of **(J)** systemic memory-like NK cells and **(K)** NK cells with VL. **(L)** Evaluation of ILC1 over the course of vaccination of female macaques. **(M, N)** Correlation of **(K)** ILC1 and **(L)** ILC2 with number of challenges. **(O, P)** Correlation of **(O)** env-reactive IFN-γ^+^ ILC1 and **(P)** env-reactive IL-17^+^ ILC3 with VL. Violin plot data shown in **(C, G, H, L)** were analyzed with Wilcoxon signed-rank test. Data shown in **(D-F, I-K, M-P)** were analyzed by the Spearman correlation test. Violin plot vertical bars denote median and quartiles. Here, black symbols represent ΔV1 SIV vaccinated female macaques.

We further evaluated memory-like NK cells, NK cells and antigen-reactive ILCs in the blood of ΔV1 SIV vaccinated female macaques (**Figures 4A, B**). We observed an increase in mucosal memory-like NK cells after vaccination in ΔV1 SIV vaccinated macaques (**Figure 4C**). These systemic cells correlated with the number of intravaginal challenges (**Figure 4D**), as has been observed for mucosal memory-like NK cells (**Figure 1F**). Interestingly, post-vaccination memory-like NK cells in mucosa and blood correlated with each other (**Figure 4E**), suggesting vaccination increases the frequency of these cells in both compartments. Furthermore, as in mucosal memory-like NK cells (**Figure 1K**), systemic memory-like NK cells also correlated with V2-specific ADCC (**Figure 4F**), further confirming the role of these cells in ADCC activity. In contrast to mucosal/systemic memory-like NK cells (**Figures 1D**, **4C**) and mucosal NK cells (**Figure 3B**), we did not observe an increase in systemic total NK cell frequency (**Supplementary Figure S6A**), suggesting total systemic NK cells react differently to vaccination. Upon stimulation with gp120-overlapping peptides, post-vaccinated memory-like NK cells expressed higher level of granzyme B (GranB) (**Figure 4G**) and IFN-γ (**Figure 4H**) compared to baseline, which was not observed for systemic total NK cells (**Supplementary Figures S6B, C**). Interestingly, compared to baseline, PMA/Ionomycin stimulation of post-vaccination PBMCs showed a trend towards an increase in granzyme B^+^ (GranB^+^) memory-like NK cells (**Supplementary Figure S6D**) and no changes for IFN-γ^+^ memory-like NK cells (**Supplementary Figure S6E**). Stimulation with PMA/Ionomycin did not change post vaccination cytokine expression compared to baseline for total NK cells (**Supplementary Figures S6F, G**). Furthermore, gp120-stimulated (**Figure 4I**) as well as PMA/Ionomycin-induced Granzyme B^+^ memory-like NK cells (**Supplementary Figure S6H**) showed a positive correlation with number of challenges. A negative correlation was observed between systemic memory-like NK cells at week 17 and VL at 1 wpi (**Figure 4J**) as well as NK cells at week 17 and VL at 1 wpi (**Figure 4K**), suggesting the role of these systemic cells in VL control. Taken together, systemic memory-like NK cells react differently to antigen stimulation (**Figures 4G, H**) compared to non-specific stimulation (**Supplementary Figures S6D, E**) and play a protective role in SIV infection.

Next, we focused on ILCs and observed that post vaccination ILC1 were significantly increased (**Figure 4L**) and ILC2 showed a trend of increase (**Supplementary Figure S6I**) compared to baseline. These cells showed a positive correlation with number of challenges (**Figures 4M, N**). However, we did not observe an increase of antigen-reactive responses of systemic ILCs (data not shown), as has been observed in the mucosal ILCs (**Figures 2E-G**). However, env-reactive IFN-γ^+^ ILC1 showed a positive correlation with VL (**Figure 4O**) and env-reactive IL-17^+^ ILC3 showed a negative correlation with VL (**Figure 4P**). Taken together, these data suggest that mucosal ILCs react differently to antigen stimulation compared to systemic ILCs, which highlights the importance of studying mucosal immune responses along with systemic responses.

### Correlation of systemic cytokines/chemokines with systemic/mucosal NK/ILCs

**Figure 5:**
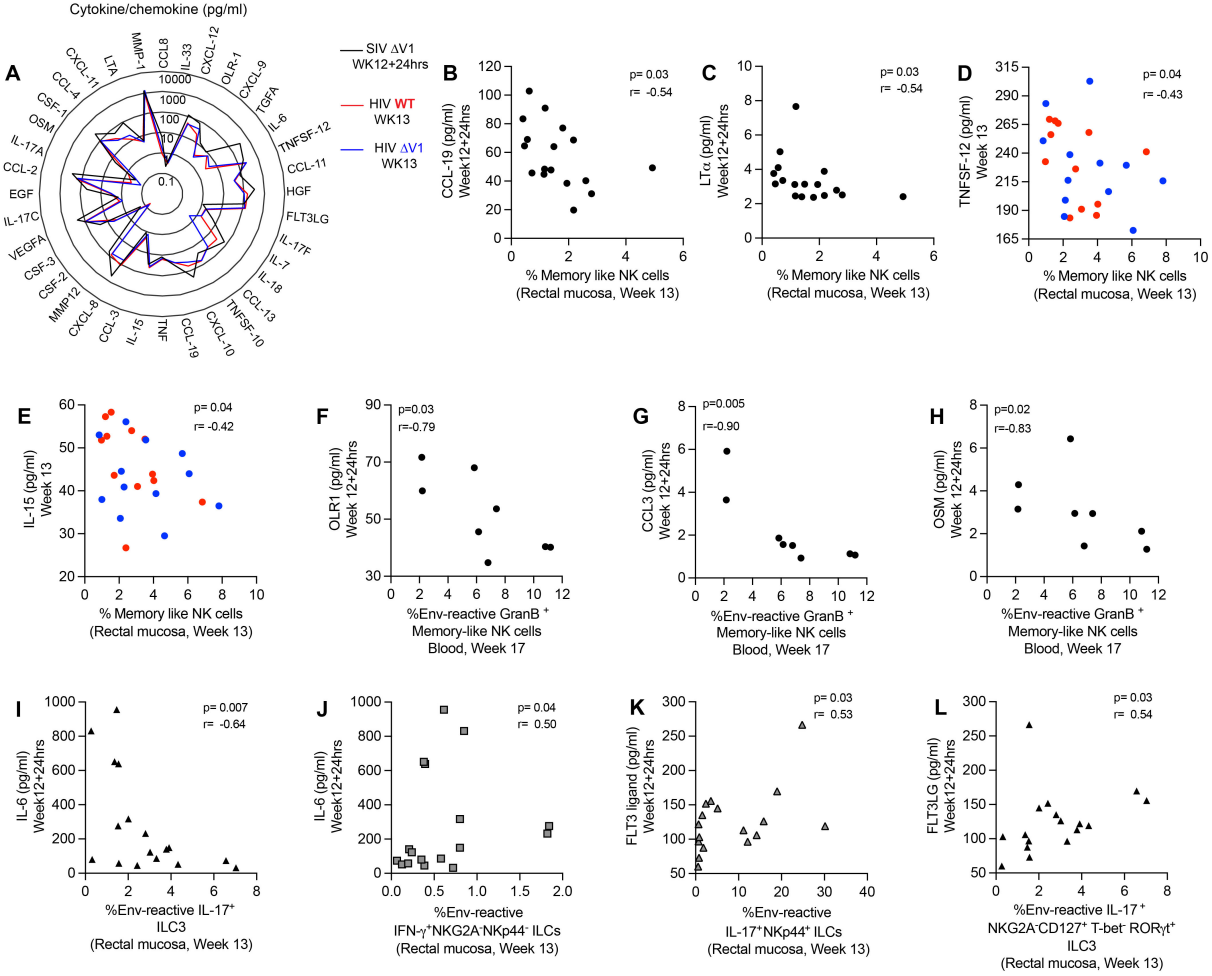
Correlation of plasma cytokines/chemokines with mucosal/systemic immune responses. **(A)** Radar plots comparing the level of 36 cytokines/chemokines in the plasma of SIV ΔV1 vaccinated animals at 24 hrs after last immunization (Week12 + 24hrs) and of HIV vaccinated animals 1 week after last immunization (Week13). **(B-E)** Correlation of mucosal memory-like NK cell frequency with **(B)** CCL-19, **(C)** LT-α, **(D)** TNFSF-12, and **(E)** IL-15. **(F-H)** Correlation of systemic env-reactive GranB^+^ memory-like NK cell frequency with **(F)** OLR-1, **(G)** CCL-3, and **(H)** OSM. **(I, J)** Correlation of IL-6 with **(I)** mucosal env-reactive IL-17^+^ ILC3 and **(J)** mucosal env-reactive IFN-γ^+^ NKG2A^-^NKP44^-^ ILCs. **(K, L)** Correlation of FLT3 ligand with **(K)** mucosal env-reactive IL-17^+^ NKp44^+^ ILCs and **(L)** mucosal env-reactive IL-17^+^ ILC3. Data for female macaques are shown in panels **(B, C, F-L)**, and data for male macaques shown in panels **(D, E).** Data shown in **(B-L)** were analyzed by the Spearman correlation test. The radar plot represents the mean value of cytokine responses. Here, black/gray, red and blue symbols represent ΔV1 SIV vaccinated female macaques, WT HIV vaccinated male macaques and ΔV1 HIV vaccinated male macaques, respectively.

In order to investigate the effect of cytokines/chemokines on NK/ILCs, we performed correlation analyses of 36 cytokines/chemokines in plasma relative to mucosal NK/ILC subsets after the last immunization. For the ΔV1 SIV vaccine, we assessed cytokines/chemokines at 12 weeks+24hrs post-immunization, and at week 13 for the HIV vaccine (**Figure 5A**). We observed a negative correlation between the frequency of mucosal memory-like NK cells (CD45^+^lineage^-^HLA-DR^-^Syk^-^Fc*ϵ*R1*γ* ^-^NKG2A^+^ cells) and CCL-19 (**Figure 5B**) and LT-α (**Figure 5C**) for the ΔV1 SIV vaccinated macaques, and between TNFSF-12 (**Figure 5D**) and IL-15 (**Figure 5E**) for HIV vaccinated macaques. Furthermore, systemic gp120-reactive GranB^+^ memory-like NK cells negatively correlated with OLR1 (**Figure 5F**), CCL-3 (**Figure 5G**) and OSM (**Figure 5H**). PMA/Ionomycin induced GranB^+^ memory-like NK cells positively correlated with IL-33 (**Supplementary Figure S7A**), CCL-13 (**Supplementary Figure S7B**) and MMP-1 (**Supplementary Figure S7C**). IL-6 correlated negatively with env-reactive IL-17^+^ ILC3 (**Figure 5I**) and positively with env- reactive IFN-γ^+^ NKG2A^-^NKp44^-^ cells (**Figure 5J**) in the ΔV1 SIV vaccinated macaques. Furthermore, in the HIV vaccinated macaques, PMA/Ionomycin induced IL-17^+^ NKp44^+^ cells also correlated negatively with TNFSF-12 (**Supplementary Figure S6D**) and IL-15 (**Supplementary Figure S6E**). The TNF level also positively associated with non-protective PMA/Ionomycin-induced CD107a^+^ NKG2A^-^NKp44^-^ cells (**Supplementary Figure S6F**) and IFN-γ^+^ NKG2A^-^NKp44^-^ cells (**Supplementary Figure S6G**). In summary, in the present study we found CCL-19, LT-α, TNFSF-12, IL-15, TNF, and IL-6 all played non-protective roles in vaccination by negatively affecting protective mucosal NK/ILC responses or by promoting non-protective mucosal NK/ILC responses. Interestingly, these cytokines have already been associated with worse outcomes in HIV infection (39–45).

Finally, we observed a positive correlation between Flt3 ligand and env-reactive IL-17^+^NKp44^+^ ILCs (**Figure 5K**) and env-reactive IL-17^+^ ILC3 (**Figure 5L**) in the ΔV1 SIV vaccinated macaques. Thus, we confirm a protective role played by Flt3 ligand, as has been observed in other studies (46, 47).

## Discussion

Epigenetic changes transform memory-like NK cells into long-lived cells with recall-like responses (14, 36). *In vitro* experiments reveal that in the presence of human cytomegalovirus (HCMV) infected cells and anti-HCMV antibodies, but not in the absence of these antibodies, these cells exhibit robust expansion, suggesting the role of antibody binding in driving their proliferation (14, 36). The adaptive nature of the “memory-like” NK cells has been extensively documented in murine (5–8), macaque (6, 9–11), and human (6, 12–14) systems. Studies on macaques infected with SIV/SHIV or vaccinated with an Ad26 platform further demonstrated antigen-reactive NK cell activity, suggesting the presence of antigen-reactive memory responses in the macaque model (9). In our current study, vaccination mediated the proliferation of memory-like NK cells (**Figures 1D, G**), with elevated frequencies persisting even 12 weeks post infection (**Figure 1D**). Consistent with the murine cytomegalovirus model where “memory-like” adaptive NK cells generate higher levels of IFN-γ compared to the non-adaptive NK cells (5), we observed an increase in env-reactive IFN-γ^+^ mucosal (**Figure 1E**) and systemic (**Figure 4H**) memory-like NK cell responses after vaccination of the ΔV1 SIV macaques. Interestingly, non-adaptive mucosal NK cells did not show any antigen-reactive IFN-γ responses (**Supplementary Figures S4B**, **S6C**), underscoring the specificity of the observed memory-like NK cells towards their corresponding antigens. In a previous study of chronically SIV infected animals, mucosal memory-like NK cells exhibited impaired cytokine production and responded to anti-gp120 antibody and gag peptides, whereas non-memory-like NK cells did not exhibit such responses (11). Additionally, decreased DAP12 activation signaling, and ZAP70 tyrosine kinase activity have been observed in memory-like NK cells post-SIV infection, indicating compromised functionality of these cells (10). Though these reports suggested that chronic SIV infection impairs memory-like NK cell activity (10, 11), our current study shows that at 12 weeks post infection, memory-like NK cells maintain expression of env-reactive IFN-γ comparable to pre-infection levels (**Figure 1E**), suggesting memory-like NK cells activity remained unimpaired three months post-SIV infection. Thus, DNA/ALVAC/gp120-based vaccination might maintain the memory-like NK cell activity even during acute SIV infection. Taken together, our findings suggest that the DNA/ALVAC/gp120-based vaccination effectively generates mucosal memory-like NK cells whose functionality remains unaffected early in infection.

V2-specific ADCC responses have been documented as a correlate of decreased risk of infection associated with the number of SIV/SHIV challenges in DNA/ALVAC/gp120-based vaccination in the macaque model (23–26, 48). In humans, CMV infection induces clonal expansion of memory-like NK cells, which express higher CD107a^+^ responses when cultured with antibody-coated Raji cells, suggesting their potential to mediate ADCC activity (27). In the present study, we observed a positive correlation between mucosal (**Figure 1K**; **Supplementary Figure S1F**) and systemic memory-like NK cells (**Figure 4F**) with V2-specific ADCC responses as well as with the number of SIV/SHIV challenges (**Figures 1F, H**, **4D**), suggesting memory-like NK cells might indeed mediate ADCC and potentially influence the vaccine efficacy outcome.

In MCMV infected mice, liver ILC1 cells showed notable transcriptional, epigenetic, and phenotypical changes, displaying enhanced protective effector responses upon secondary MCMV challenges (15). Furthermore, in hapten sensitized mice, hapten-specific IL-7Rα^+^memory ILC1 cells were generated in both LN and liver (16), suggesting MCMV infection or hapten immunization were able to generate memory ILC1 responses. In the murine lung model, exposure to allergens or IL-33 induces expansion of lung ILC2 populations, and a subset persists even after inflammation resolves. Upon secondary exposure to unrelated allergens, these long-lived cells exhibit higher reactivity (17). Furthermore, transfer of ILC2 from the chronic asthma mouse model to naïve mice established chronic asthma in the recipients (18). Together these reports suggested a memory-like property of ILC2. A memory property for ILC3 was similarly suggested by results from the murine model. Enterobacterial challenge to mice generates long-term ILC3 activation, and upon subsequent rechallenge these “trained” cells proliferate, express enhanced IL-22, and exhibit superior infection control compared to naïve ILC3 (19).

In the current study, we observed expansion of mucosal ILC1, ILC2, and ILC3 (**Figures 2B-D**; **Supplementary Figures S2A-C**), and systemic ILC1 (**Figure 4L**) populations upon vaccination with the mucosal cell frequency increasing further 12 weeks post infection (**Figures 2B-D**). We next looked for antigen reactivity of these cells and found that upon overlapping env peptide stimulation, antigen-reactive mucosal IFN-γ^+^ILC1, IL-13^+^ILC2, and IL-17^+^ ILC3 frequency also increased in the ΔV1 SIV macaques (**Figures 2E-G**). No such changes were observed for systemic ILCs. Furthermore, in these same macaques mucosal env-reactive IL-13^+^ILC2 and IL-17^+^ ILC3 cells correlated positively with the number of viral challenges (**Figures 2I, J**), suggesting a protective role against SIV infection. However, no such systemic antigen-reactive ILC expansion was observed upon vaccination. Thus, we found that the vaccination regimens described here were able to generate mucosal ILC1, ILC2, ILC3 subsets with memory recall properties, and that the antigen reactive ILCs played a role in protecting macaques from SIV infection.

Flagellin-induced inflammation in mice increases the frequency of ILC3 patrolling the mucosa, which helps maintain intestinal barrier integrity (49). Furthermore, IL-17 contributes to the formation of tight junctions in the gut epithelium to preserve mucosal integrity (50), potentially making the gut mucosa less susceptible to SIV infection. In this study, we observed that NKp44^+^ cell frequency increased upon vaccination (**Figure 3C**; **Supplementary Figure S4E**), and this was associated with reduced risk of infection (**Figures 3D, E**). We further observed that, with vaccination, these cells showed increased env-reactive IL-17 responses (**Figure 3H**). Mucosal NKp44^+^ ILCs expressing env- reactive IL-17 have already been associated with protection from SIV/SHIV infection (11, 23, 26, 35). Thus, our vaccination strategy in the current study was able to generate antigen-reactive “memory-like” NKp44^+^ ILCs to protect against SIV infection.

The members of the TNF superfamily are proinflammatory cytokines (51) and play a role in HIV pathogenesis (39). Lymphotoxin-alpha (LT-α) is one such cytokine (39). Elevated plasma levels of IL-15, another pro-inflammatory cytokine, have been observed in individuals with HIV-1 infection (52) and are also correlated with higher HIV viral load (40–42) due to their association with the frequency of HIV-1 target cells (53, 54). CCL-19 promotes inflammation among HIV-1 infected patients (55), and is associated with viral integration and the establishment of latent HIV reservoirs within CD4 T cells (43, 44). OLR1 (Oxidized Low Density Lipoprotein Receptor 1) has been reported to be elevated in HIV infected individuals (56). CCL3 is one of the natural ligands for HIV coreceptor CCR5 and might play a protective role against HIV infection (57), however, higher *in vitro* CCL3 (MIP1a) in the culture supernatant was associated with HIV infection (58). Oncostatin M (OSM), a member of the interleukin-6 cytokine family, which might facilitate HIV infection (45). In the current study, these inflammatory cytokines showed a negative correlation with the frequency of protective memory-like NK cells (**Figures 5B-E**) and env-reactive GranB^+^ memory-like NK cells (**Figures 5F-H**). Furthermore, IL-33 regulates immune responses and might play a role against HIV infection (59). The role of CCL-13 and MMP-1 in HIV infection is not well understood (60, 61). These cytokines positively correlated with PMA/Ionomycin -induced GranB^+^ memory-like NK cells (**Supplementary Figures S7A-C**). Furthermore, TNF levels positively correlated with non-protective cytokine-expressing NKG2A^-^NKp44^-^ cells (**Supplementary Figure S7F**) or exhibited a similar trend (**Supplementary Figure S7G**). IL-6 levels have been associated with HIV viremia (45). In the current study, IL-6 negatively correlated with protective IL-17^+^ ILCs (**Figure 5I**). Consistent with the previously described characteristics of these cytokines/chemokines, we found that CCL-19, LT-α, TNFSF-12, IL-15, TNF, and IL-6 all suppressed the protective mucosal NK/ILC responses. Conversely, Flt3 ligand enhances the immunogenicity of HIV peptide vaccines (46) and suppresses HIV infection in humanized mice (47). As expected, in our study Flt3 ligand showed a positive correlation with protective IL-17^+^ ILCs (**Figures 5K, L**), indicating its protective role.

A limitation of this study was that the female macaques were challenged intravaginally, and immune responses were determined in the rectal mucosal tissue. We avoided vaginal tissue collection prior to virus challenge exposure since it takes longer to heal than rectal mucosa. A similar limitation did not apply to male macaques however, as both the assessment of immune responses and SHIV challenges exposure were conducted on rectal mucosa. An earlier study showed that the balance of NKp44^+^ ILCs and DN ILCs differs between rectal and vaginal tissue, but nevertheless rectal immune responses correlated with vaginal challenge outcome (11). Accordingly, rectal memory-like NK cells also correlated with reduced risk of vaginal SIV acquisition in our studies, as well as with risk of rectal SHIV acquisition. Furthermore, we demonstrate that rectal antigen-reactive IL-17^+^ ILC3, NKp44^+^ ILC and IL-17^+^ NKp44^+^ ILC also correlated with reduced risk of vaginal as well as rectal challenges. Although the frequency and dynamics of NK/ILC may vary between vaginal and rectal mucosa, the finding of interchangeable immune correlates in the two compartments suggests a functional commonality.

**Figure 6:**
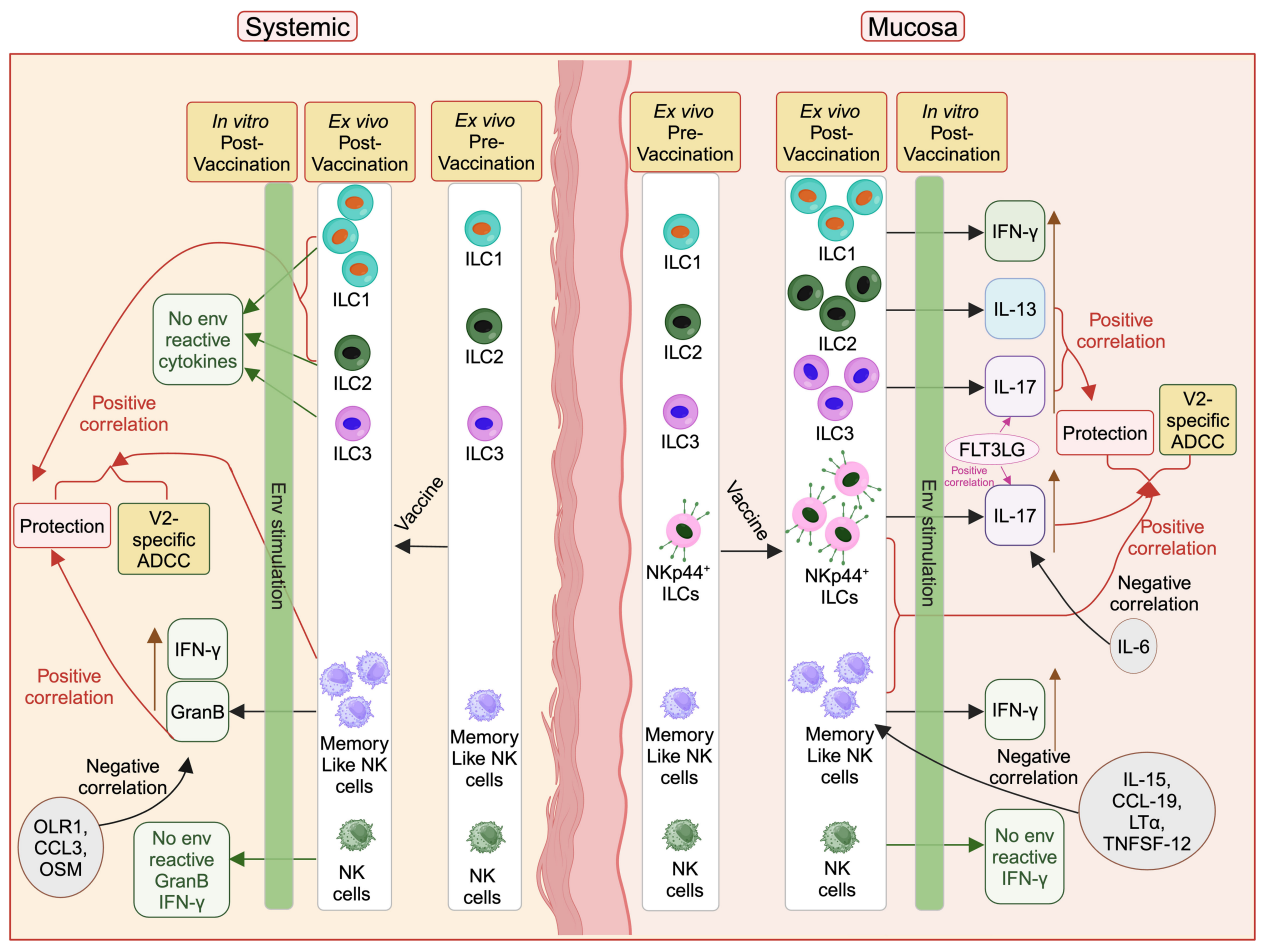
Protective role of vaccine induced mucosal/systemic NK/ILC in SIV/SHIV infection. Administration of vaccine increases different NK/ILC populations in the mucosa as well as in blood. Mucosal env-reactive IL-17^+^ NKp44^+^ ILCs, env-reactive IL-17^+^ ILC3, env-reactive IL-13^+^ ILC2, NKp44^+^ ILCs, and memory-like NK cells, as well as systemic memory-like NK cells, ILC1 and ILC2 showed a protective role against SIV/SHIV infection. IL-15, CCL-19, LT-α, and TNFSF-12 cytokines suppress the mucosal memory-like NK cells and OLR1, CCL3 and OSM suppress systemic GranB^+^ memory-like NK cells. IL-6 suppresses protective mucosal env-reactive IL-17^+^ NKp44^+^ ILCs responses. FLT3 ligand facilitate protective mucosal env-reactive IL-17^+^ NKp44^+^ ILCs and env-reactive IL-17^+^ ILC3 responses. This figure was created with BioRender.com.

We show for the first time that DNA/ALVAC-based vaccination can generate mucosal/systemic memory-like NK cells as well as mucosal antigen-reactive ILCs (**Figure 6**). Importantly, the memory-like NK cells induced by this platform were associated with reduced risk of SIV/SHIV infection and also correlated with V2-specific ADCC, one of the primary correlates of protection induced by the DNA/ALVAC/gp120 vaccine regimen (23–25). Further, we showed that vaccination induced pro-inflammatory antigen-reactive mucosal not systemic ILC responses (**Figure 6**) in the form of vaccine-induced env-reactive IL-13^+^ ILC2 (**Figure 2F**), IL-17^+^ ILC3 (**Figure 2G**), and IL-17^+^ NKp44^+^ ILC (**Figure 3G**), which were associated with reduced risk of SIV infection. Furthermore, post-vaccination systemic cytokines/chemokines were associated with the frequency of mucosal/systemic NK/ILC populations and likely affected the balance of protective and non-protective responses. In addition to identifying the role of NK subsets in the protection afforded by the V1-deleted DNA/ALVAC platform, our findings suggest that adaptive “memory-like” NK cells and antigen- reactive ILC2, ILC3, and NKp44^+^ ILCs contribute to the efficacy of this vaccine approach. Engagement of NK/ILC subsets may prove to be essential for a fully effective vaccine against HIV.

## Materials and methods

### Animals

Eighteen female and twenty-four male Indian rhesus macaques obtained from the free-range breeding colony on Morgan Island, South Carolina, were used in this study. The macaques, aged 2 to 5 years at study initiation, were negative for SIV, simian retrovirus, and STLV. Macaques were housed and maintained at the NCI Animal Facility at the National Institutes of Health, Bethesda, MD. All animals were handled in accordance with the standards of the Association for the Assessment and Accreditation of Laboratory Animal Care (AAALAC) in an AAALAC-accredited facility (OLAW, Animal Welfare Assurance A4149-01 for NIH). All animal care and procedures were carried out under protocols approved by the NCI Animal Care and Use Committee (ACUC) prior to study initiation. Animals were closely monitored daily for any signs of illness, and appropriate medical care was provided as needed. Animals were socially housed per the approved ACUC protocol and social compatibility except during the viral challenge phase when they were individually housed. All clinical procedures, including biopsy collection, administration of anesthetics and analgesics, and euthanasia, were carried out under the direction of a laboratory animal veterinarian. Steps were taken to ensure the welfare of the animals and minimize discomfort of all animals used in this study. Animals were fed daily with a fresh diet of primate biscuits, fruit, peanuts, and other food items to maintain body weight or normal growth. Animals were monitored for psychological well-being and provided with physical enrichment including sanitized toys, destructible enrichment (cardboard and other paper products), and audio and visual stimulation.

### Immunization and challenge

Eighteen female macaques (ΔV1 SIV group) were MHC typed for Mamu A*01, Mamu B*17 and Mamu B*08; three of them were Mamu A*01 positive and one was B*17 positive. The animals were immunized at weeks 0 and 4 with *SIV_766_ gp160ΔV1 DNA* (2 mg/dose) and *SIV_mac239_ p57 Gag DNA* (1 mg/dose) in a total volume of 1 ml PBS. The DNA was administered in both thighs (0.5 ml to each). At 8 weeks the macaques were administered ALVAC-SIV encoding gag/pro/gp120-TM (vCP2432-wildtype env) in the right thigh, 10^8^ pfu/dose in 1 ml PBS. At week 12, the macaques were boosted with the same ALVAC-SIV plus SIV gp120ΔV1 protein (400 µg/dose in 500 ml PBS plus 500 ml 2% Alhydrogel). The ALVAC-SIV was administered to the right thigh; the 1 ml dose of Env protein plus alum was administered to the left thigh. Beginning at week 17 all macaques were challenged intravaginally weekly with 1 ml of a SIV_mac251_ stock containing 800 median tissue culture infectious doses (TCID50). Up to 14 challenges were administered until the macaques became SIV positive. Infection was determined by analysis of plasma for viral loads >50 SIV RNA copies/ml plasma assessed weekly by the droplet digital PCR method. One week after every challenge the plasma was collected, and PCR was performed to determine the VL. The infected animals were followed upto 15 weeks for VL post-infection.

For SIVmac251 challenge 2010 Day 8 virus to challenge stock was used (62). Virus titers were performed on primary rhesus macaque cells (63). Briefly, CD8-depleted PBMCs from 3 SIV-naïve rhesus macaques were stimulated with plate-bound anti-CD3 for 3 days, individually plated at 10^5^ cells/well in 96-well plates and infected in triplicate with serial 5-fold dilutions of virus. Cells were washed 3 times with PBS 24 h postinfection to remove residual input virus and were then maintained in 100 U/ml IL-2 for 3 weeks. Cell-free culture supernatants were then collected, and a SIV p27 antigen capture assay was used to detect the presence of viral p27 antigen according to the manufacturer’s instructions (ABL). The 50% tissue culture infectious dose (TCID_50_) was then calculated using the Reed-Muench accumulative method (63).

Twenty-four male macaques were used in another arm of the study, they were not subtyped for Mamu and was randomized based on age and weight. Twelve male macaques (WT HIV group) were immunized at weeks 0 and 4 with HIV *A244 gp160 DNA* (2 mg/dose) and HIV BIIILAI *p55 Ga*g *DNA*(1 mg/dose) in a total volume of 1 ml PBS. The DNA was administered in both thighs (0.5 ml to each). At 8 weeks the macaques were administered ALVAC-HIV encoding *gag/pro/gp120-TM* (vCP2438 - wildtype *env*) in the right thigh, 10^8^ pfu/dose in 1 ml PBS. At week 12 the macaques were boosted with the same ALVAC-HIV plus HIVgp120 protein (400 µg/dose in 700 ml PBS plus 700 ml 2% Alhydrogel). The ALVAC-HIV was administered to the right thigh; the 1.4 ml dose of Env protein plus alum was administered to the left thigh. Another twelve male macaques (ΔV1 HIV group) were immunized at weeks 0 and 4 with HIV A244 *gp160ΔV1DNA* (2 mg/dose) and HIV BIIILAI *p55 Gag DNA* (1 mg/dose) in a total volume of 1 ml PBS. The DNA was administered in both thighs (0.5 ml to each). At 8 weeks the macaques were administered ALVAC-HIV encoding *gag/pro/gp120TM* (vCP2438 – wildtype *env*) in the right thigh, 10^8^ pfu/dose in 1 ml PBS. At week 12 the macaques were boosted with the same ALVAC-HIV plus HIV gp120ΔV1 protein (400 µg/dose in 700 ml PBS plus 700 ml 2% Alhydrogel). The ALVAC was administered to the right thigh; the 1.4 ml dose of Env protein plus alum was administered to the left thigh. Beginning at week 21, all 24 male macaques were challenged intrarectally weekly with 1 ml of a SHIV_157(QNE)Y173H_ diluted 1:10,000 from stock. Up to 11 challenges were administered until the macaques became SHIV positive. Infection was determined by analysis of plasma for viral loads >62 SHIV RNA copies/ml plasma assessed weekly by the droplet digital PCR method. One week after every challenge the plasma was collected, and PCR was performed to determine the VL. The infected animals were followed upto 15 weeks for VL post-infection.

The SHIV-1157(QNE)Y173H virus stock was grown from the infectious molecular clone in rhesus peripheral blood mononuclear cells (PBMCs) and the stock was titrated in rhesus macaques to select the appropriate dilution (64) and animals were challenged by the intrarectal route with 1:10,000 dilution (64). In this study the published protocol was followed.

### SIV viral RNA by droplet PCR

Digital Droplet PCR (ddPCR) is a novel platform designed to provide great sensitivity, accuracy and precision for the detection and absolute quantitation of nucleic acid target molecules in a sample. The system partitions the small amount (20 μL) of the RNA extract volume into 20,000 nL droplets. The droplets contain a random distribution of both the target and/or background RNA molecules. The enzymes, reverse transcriptase (RT) and Taq DNA polymerase (Bio-Rad one-step ddPCR kit) blend into the droplets and are inactive until the reverse transcription reaction is performed at 50°C. SIV gag forward primer: 5’-GCAGAGGAGGAAATTACCCAGTAC-3’, SIV gag reverse primer: 5’- CAATTTTACCCAGGCATTTAATGTT-3’ were used for the reaction. Double-quenched SIV gag probe: 5’-/56FAM/TGTCCACCTGCCATTAAGCCCGA-3’ were included at the start of the reaction in each droplet. Each droplet (1nL) is defined on the based-on fluorescence amplitude as being either positive or negative. Prior to droplet generation, nucleic acid samples are prepared and mixed with primers and fluorescent probes, and Supermix (One-Step RT-ddPCR Advanced Kit; BioRad Catalog No. 1864022) to create to create up to 45 prepared samples including positive and negative controls for droplet generation in a 96-well PCR plate. After droplets are transferred into the 96-well plate, PCR amplification is performed to endpoint with the PCR thermal cycler. The plate is then transferred to a beta-prototype droplet reader (optical reader) that employs an integrated autosampler and fluidics to serially aspirate droplets from each well and stream them single-file, at a rate of about 1500 droplets/second, past a two-color fluorescence detector sampled at a rate of 100 kHz on both FAM and HEX fluorescence channels. Poisson statistics are used to quantify the proportion of positive droplets, i.e., the number of target templates from which absolute viral RNA levels can be calculated precisely in copies/µL. The fraction of fluorescent droplets determines the concentration of the target in the sample. Copies/mL were calculated based on copies/µL (Volume of MM per well/Volume of template) * plasma dilution factor/(volume of plasma used for extraction/elution volume). The assay’s limit of quantification (LOQ) is set at 50 RNA copies per milliliter of plasma.

### SHIV viral RNA by droplet PCR

SHIV 1157 QNE (Y173H) RNA copies per milliliter were determined by a two-step real time qPCR (65), performed by NHP Core Virology Laboratory at Duke University using -80°C frozen EDTA plasma samples. Briefly, Viral RNA was extracted using an automated sample preparation platform QIA symphony SP (Qiagen, Hilden, Germany) along with a Virus/Pathogen DSP midi kit and the *cellfree500*protocol. The extracted RNA was annealed with a reverse primer designed specifically for the gag gene of SIVmac251 (5′-CAC TAG GTG TCT CTG CAC TAT CTG TTT TG-3′) (SHIV 1157 QNE (Y173H) and SIVmac251 shares the gag gene sequence). Subsequently, SuperScript™ III Reverse Transcriptase (Thermo Fisher Scientific, Waltham, MA, USA) alongside RNAse Out (Thermo Fisher Scientific), were used to reverse transcribe RNA into cDNA. The cDNA was treated with RNase H (Thermo Fisher Scientific) and introduced (in duplicate) into a customized 4x TaqMan™ Gene Expression Master Mix (Thermo Fisher Scientific), which contained primers, and a hydrolysis probe labeled with fluorescence and tailored specifically for the gag gene of SIVmac251. The primer set included a forward primer (5′-GTC TGC GTC ATC TGG TGC ATT C -3′) and a reverse primer identical to the one used for annealing. Additionally, the mix comprised a fluorescently labeled hydrolysis probe (5′-/56-FAM/CTT CCT CAG TGT GTT TCA CTT TCT CTT CTG CG/3BHQ_1/-3′). The qPCR was performed on a StepOnePlus™ Real-Time PCR System (Thermo Fisher Scientific), applying the subsequent thermal cycling conditions: initial denaturation at 50°C for 2 minutes, followed by an initial activation step at 95°C for 10 minutes. The cycling process was then repeated for 50 cycles, 95°C for 15 seconds, to cool to 60°C for 1 minute. Mean copies of SIV gag RNA per reaction were computed by interpolating quantification cycle data against a serial dilution of a characterized custom RNA transcript encompassing a 730 bp segment of the SIV gag gene. Mean RNA copies per milliliter were determined by factoring in the assay dilution factor (DF = 18.72). The assay’s limit of quantification (LOQ) is set at 62 RNA copies per milliliter of plasma.

### Mucosal NK/ILC phenotyping in rectal tissue of macaques

The frequency of NK/ILCs was measured in the rectal mucosa of the female ΔV1 SIV group of macaques pre vaccination, 1 week post prime (week 5), 1 week post last vaccination (week 13), and 12 weeks post infection (12 wpi). Measurements were made in the HIV and ΔV1 subgroups of the male macaques pre vaccination and 1 week post last vaccination (week 13). Freshly collected rectal biopsies were processed to the single cell level. A portion of the cells was used for phenotyping. Subsequently, cells were stained with Blue Dye (cat. #L34962, 0.5 μl) from Thermo Fisher, followed by surface staining with the following: Alexa 700 anti-CD3 (SP34-2; cat. #557917, 5 μl), Alexa 700 anti-CD20 (2H7; cat. #560631, 5 μl), BV421 anti-CD14 (M5S2; cat. #558121, 5 μl), BUV661 anti-HLA-DR (G46-6; cat. #612980, 5 μl), APC-Cy7 anti-CD11b (ICRF44; cat. # 557754, 5 μl), BV650 anti-NKp44 (P44-8; cat. # 744302, 5 μl), BV786 anti-CD45 (D058-1283; cat. # 563861, 5 μl) from BD Biosciences (San Jose, California, USA); PE-Cy7 anti-NKG2A (Z199; cat. no. B10246, 5 μl) from Beckman Coulter; and PE-Cy5 anti-CD127 (A019D5; cat. # 351324, 5 μl), from Biolegend for 30 minutes at room temperature. This was followed by permeabilization with a FOX3-transcription buffer set (cat. #00-5523-00) from eBioscience (San Diego, California, USA) according to the manufacturer’s recommendation. Intracellular staining was subsequently performed with the following: BV605 anti-T-bet (4B10; cat. # 644817, 5 μl), from Biolegend; PE anti-ROR gamma(t) (AFKJS-9; cat. # 12-6988-82, 5 μl), APC anti-Syk (4D10.1; cat. #17-6696-42, 5 μl) from Thermo Fisher, and FITC Anti-FcϵRI gamma-chain (cat. #FCABS400F, 5 μl) from Millipore Sigma for 30 minutes at room temperature. Samples were acquired on a BD FACSymphony A5 cytometer and analyzed with FlowJo software 10.6. Memory-like NK cells were defined as CD45^+^ lineage^-^ HLA-DR^-^ Syk^-^ γ-chain (Fc*ϵ*R1*γ*)^-^ NKG2A^+^ cells (10, 11). ILC1were defined as CD45^+^lineage^-^NKG2A^-^ T-bet^+^ cells, ILC2 as CD45^+^lineage^-^NKG2A^-^ T-bet^-^RORγt^-^ cells and ILC3 as CD45^+^lineage^-^NKG2A^-^ T-bet^-^RORγt^+^ cells (30, 31, 37). CD45^+^lineage^-^ NKG2A^+^ cells were defined as NK cells (38). NKp44 ILCs were gated as CD45^+^lineage^-^ NKp44^+^ cells, NKG2A^-^NKp44^-^ double negative ILCs were gated as CD45^+^lineage^-^ NKG2A^-^NKp44^-^ cells (11, 23, 35).

### Intracellular antigen-reactive/non- reactive cytokine expression by mucosal NK/ILC

The cytokine expression of NK/ILCs was measured in macaque rectal mucosa tissue pre vaccination, 1 week post prime (week 5), 1 week post last vaccination (week 13) and 12 weeks post infection for the ΔV1 SIV female macaque group and pre vaccination and 1 week post last vaccination (week 13) for the HIV and ΔV1 HIV groups of male macaques. Freshly collected rectal biopsies were processed and were stimulated with overlapping gp120 peptides (2 μg/ml) or PMA/Ionomycin for 2 hours. Subsequently, GolgiPlug protein transport inhibitor (containing Brefeldin A) (cat. #555029, 1 μl) and GolgiStop protein transport inhibitor (containing Monensin) (cat. #554724, 0.7 μl) were added and culturing continued for 18 hours. Subsequently, cells were stained with Blue Dye (cat. #L34962, 0.5 μl) from Thermo Fisher, followed by surface staining with the following: Alexa 700 anti-CD3 (SP34-2; cat. #557917, 5 μl), Alexa 700 anti-CD20 (2H7; cat. #560631, 5 μl), BV421 anti-CD14 (M5S2; cat. #558121, 5 μl), BUV661 anti-HLA-DR (G46-6; cat. #612980, 5 μl), APC-Cy7 anti-CD11b (ICRF44; cat. # 557754, 5 μl), BV650 anti-NKp44 (P44-8; cat. # 744302, 5 μl), BV786 anti-CD45 (D058-1283; cat. # 563861, 5 μl) from BD Biosciences (San Jose, California, USA); PE-Cy7 anti-NKG2A (Z199; cat. no. B10246, 5 μl) from Beckman Coulter; and PE-Cy5 anti-CD127 (A019D5; cat. # 351324, 5 μl), from Biolegend for 30 minutes at room temperature. This was followed by permeabilization with a FOX3-transcription buffer set (cat. #00-5523-00) from eBioscience (San Diego, California, USA) according to the manufacturer’s recommendation.

In female macaques, intracellular staining was performed using: BV605 anti-T-bet (4B10; cat. # 644817, 5 μl), PE-Cy5.5 anti-IL-17 (BL168; cat. # 512314, 5 μl) from Biolegend; PE anti-ROR gamma(t) (AFKJS-9; cat. # 12-6988-82, 5 μl), APC anti-Syk (4D10.1; cat. #17-6696-42, 5 μl) from Thermo Fisher, FITC Anti-FcϵRI gamma-chain (cat. #FCABS400F, 5 μl) from Millipore Sigma; BV711 anti-IL-13 (JES10-5A2; cat. # 564288, 5 μl), and PE-CF594 anti-IFN-g (B27; cat. # 562392, 5 μl) from BD Biosciences (San Jose, California, USA) for 30 minutes at room temperature. Samples were acquired on a BD FACSymphony A5 cytometer and analyzed with FlowJo software 10.6.

In male macaques, intracellular staining was performed using: BV605 anti-T-bet (4B10; cat. # 644817, 5 μl), PE-Cy5.5 anti-IL-17 (BL168; cat. # 512314, 5 μl) from Biolegend; PE anti-ROR gamma(t) (AFKJS-9; cat. # 12-6988-82, 5 μl), APC anti-Syk (4D10.1; cat. #17-6696-42, 5 μl) from Thermo Fisher, FITC Anti-FcϵRI gamma-chain (cat. #FCABS400F, 5 μl) from Millipore Sigma; BUV395 anti-CD107 (H4A3; cat. # 565113, 5 μl), BV750 anti-TNFa (MAb11; cat. # 566359, 5 μl), and PE-CF594 anti-IFN-g (B27; cat. # 562392, 5 μl) from BD Biosciences (San Jose, California, USA) for 30 minutes at room temperature. Samples were acquired as for the female macaques.

### Systemic NK/ILC phenotyping in PBMC of macaques

The frequency of NK/ILCs was measured in the PBMC of the female ΔV1 SIV group of macaques pre vaccination and 5 week post last vaccination (week 17). A portion of the cells was used for phenotyping. Cells were stained with Blue Dye (cat. #L34962, 0.5 μl) from Thermo Fisher, followed by surface staining with the following: Alexa 700 anti-CD3 (SP34-2; cat. #557917, 5 μl), Alexa 700 anti-CD20 (2H7; cat. #560631, 5 μl), BUV805 anti-CD14 (M5S2; cat. #612902, 5 μl), BUV661 anti-HLA-DR (G46-6; cat. #612980, 5 μl), APC-Cy7 anti-CD11b (ICRF44; cat. # 557754, 5 μl), BV786 anti-CD45 (D058-1283; cat. # 563861, 5 μl) from BD Biosciences (San Jose, California, USA); PE-Cy7 anti-NKG2A (Z199; cat. no. B10246, 5 μl) from Beckman Coulter; and PE-Cy5 anti-CD127 (A019D5; cat. # 351324, 5 μl), from Biolegend for 30 minutes at room temperature. This was followed by permeabilization with a FOX3-transcription buffer set (cat. #00-5523-00) from eBioscience (San Diego, California, USA) according to the manufacturer’s recommendation. Intracellular staining was subsequently performed with the following: BV605 anti-T-bet (4B10; cat. # 644817, 5 μl), from Biolegend; PE anti-ROR gamma(t) (AFKJS-9; cat. # 12-6988-82, 5 μl), APC anti-Syk (4D10.1; cat. #17-6696-42, 5 μl) from Thermo Fisher, and FITC Anti-FcϵRI gamma-chain (cat. #FCABS400F, 5 μl) from Millipore Sigma for 30 minutes at room temperature. Samples were acquired on a BD FACSymphony A5 cytometer and analyzed with FlowJo software 10.6. Memory-like NK cells were defined as CD45^+^ lineage^-^ HLA-DR^-^ Syk^-^ γ-chain (Fc*ϵ*R1*γ*)^-^ NKG2A^+^ cells (10, 11). ILC1were defined as CD45^+^lineage^-^NKG2A^-^ T-bet^+^ cells, ILC2 as CD45^+^lineage^-^NKG2A^-^ T-bet^-^RORγt^-^ cells and ILC3 as CD45^+^lineage^-^NKG2A^-^ T-bet^-^RORγt^+^ cells (30, 31, 37). CD45^+^lineage^-^ NKG2A^+^ cells were defined as NK cells (38).

### Intracellular antigen-reactive/non- reactive cytokine expression by systemic NK/ILC

The cytokine expression of NK/ILCs was measured in macaque rectal mucosa tissue pre vaccination and 5 week post last vaccination (week 17) for the ΔV1 SIV female macaque group. PBMCs were stimulated with overlapping gp120 peptides (2 μg/ml) or PMA/Ionomycin for 2 hours. Subsequently, GolgiPlug protein transport inhibitor (containing Brefeldin A) (cat. #555029, 1 μl) and GolgiStop protein transport inhibitor (containing Monensin) (cat. #554724, 0.7 μl) were added and culturing continued for 18 hours. Subsequently, cells were stained with Blue Dye (cat. #L34962, 0.5 μl) from Thermo Fisher, followed by surface staining with the following: Alexa 700 anti-CD3 (SP34-2; cat. #557917, 5 μl), Alexa 700 anti-CD20 (2H7; cat. #560631, 5 μl), BUV805 anti-CD14 (M5S2; cat. #612902, 5 μl), BUV661 anti-HLA-DR (G46-6; cat. #612980, 5 μl), APC-Cy7 anti-CD11b (ICRF44; cat. # 557754, 5 μl), BV786 anti-CD45 (D058-1283; cat. # 563861, 5 μl) from BD Biosciences (San Jose, California, USA); PE-Cy7 anti-NKG2A (Z199; cat. no. B10246, 5 μl) from Beckman Coulter; and PE-Cy5 anti-CD127 (A019D5; cat. # 351324, 5 μl), from Biolegend for 30 minutes at room temperature. This was followed by permeabilization with a FOX3-transcription buffer set (cat. #00-5523-00) from eBioscience (San Diego, California, USA) according to the manufacturer’s recommendation. Intracellular staining was performed using: BV605 anti-T-bet (4B10; cat. # 644817, 5 μl), PE-Cy5.5 anti-IL-17 (BL168; cat. # 512314, 5 μl) from Biolegend; PE anti-ROR gamma(t) (AFKJS-9; cat. # 12-6988-82, 5 μl), APC anti-Syk (4D10.1; cat. #17-6696-42, 5 μl) from Thermo Fisher, FITC Anti-FcϵRI gamma-chain (cat. #FCABS400F, 5 μl) from Millipore Sigma; BV711 anti-IL-13 (JES10-5A2; cat. # 564288, 5 μl), BV421 anti-IFN-γ (B27; cat. # 562988, 5 μl), BV570 anti-GranB (GB11; cat. # 563398, 5 μl), from BD Biosciences (San Jose, California, USA) for 30 minutes at room temperature. Samples were acquired on a BD FACSymphony A5 cytometer and analyzed with FlowJo software 10.6.

### CD4^+^ T-cell phenotyping in PBMC

The levels of CD4^+^ T-cell subsets were measured in blood at baseline and week 13 in ΔV1 SIV vaccinated female macaques (23). PBMCs were stained with the following: LIVE/DEAD Fixable Blue Dead Cell Stain (cat. no. L23105, Thermo Fisher); Alexa 700 anti-CD3 (SP34-2; cat. no. 557917, 5 μl), BV785 anti-CD4 (L200; cat. no. 563914, 5 μl), PeCy5 anti-CD95 (DX2; cat. no. 559773, 5 μl), BV650 anti-CCR5 (3A9; cat. no. 564999, 5 μl), BUV496 anti-CD8 (RPA-T8; cat. no. 564804, 5 μl), and FITC anti-Ki67 (B56; cat. no. 556026, 5 μl) from BD Biosciences; APC Cy7 anti-CXCR3 (G025H7; cat. no. 353722, 5 μl) and BV605 anti-CCR6 (G034E3; cat. no. 353420, 5 μl) from BioLegend; and APC anti-α4β7, provided by the NIH Nonhuman Primate Reagent Resource (R24 OD010976, and NIAID contract HHSN272201300031C). Samples were acquired on a BD FACSymphony A5 cytometer and analyzed with FlowJo software 10.6. Gating was done on live CD3^+^CD4^+^ cells and on vaccine induced Ki67^+^ cells. CXCR3 and CCR6 expression were used to identify Th1(Live cells/CD3^+^/CD4^+^/Ki67^+^/CD95^+^/CCR6^-^CXCR3^+^), Th2 (Live cells/CD3^+^/CD4^+^/Ki67^+^/CD95^+^/CCR6^-^CXCR3^-^) or Th17 (Live cells/CD3^+^/CD4^+^/Ki67^+^/CD95^+^/CCR6^+^CXCR3^-^) populations (4).

### Inhibition of ADCC CEM-based assay by monoclonal F(ab’)2 of NCI05 and CH58

V2-specific ADCC activity was assessed as previously described (11, 23, 24, 66) using EGFP-CEM-NKr-CCR5-SNAP cells that constitutively express GFP as targets (67). F(ab’)2 fragments were prepared from both NCI05 and CH58 mAbs, as these antibodies recognize overlapping, conformationally distinct V2 epitopes (25), using Pierce f(ab’)2 Micro Preparation Kit (cat. #44688, Thermo Fisher) following the manufacturer’s instructions. An SDS-page gel with the recovered F(ab’)2 was run and Silver stained (cat. #LC6070, Silver Quest staining Kit, Invitrogen, Waltham, Massachusetts, USA) according to the manufacturer’s instructions, to assure the purity of the F(ab’)2 fragments. Briefly, one million target cells were incubated with 50 μg of ΔV1 gp120 protein for 2 h at 37°C. After this coating, the target cells were washed and labeled with SNAP-Surface^®^ Alexa Fluor^®^ 647 (New England Biolabs, Ipswich, Massachusetts, USA) per manufacturer recommendations for 30 min at RT. 5,000 target cells (50 μl) were incubated for 1h at 37°C with 5 μg/ml of purified F(ab’)2 fragments (50 μl) from NCI05 or CH58 monoclonal antibodies in a V-bottom plate (Millipore Sigma, St. Louis, Missouri, USA). 5,000 target cells (50 μl) incubated with R10 (50 μl) without F(ab’)2 served as control. Plasma samples, heat inactivated at 56°C for 30 min, were diluted 1:250 and 50 μl were added to wells of 96-well V-bottom plate (Millipore Sigma, St. Louis, Missouri, USA). 250,000 human PBMCs (50 μl) were added as effectors to each well to give an effector/target (E/T) ratio of 50:1. The plate was incubated at 37°C for 2h followed by two PBS washes. The cells were resuspended in 200 μl of a 1% PBS– paraformaldehyde solution and a BD FACSymphony A5 cytometer and analyzed with FlowJo software 10.6. Specific killing was measured by loss of GFP from the SNAPAlexa647^+^ target cells. Target and effector cells cultured in the presence of R10 medium were used as background. Normalized percent killing was calculated using the formula: (killing in the presence of plasma or plasma + F(ab′)2 − background)/(killing in the presence of positive control − background) x100. The V2-specific ADCC killing was calculated using the formula: ADCC killing measured in the absence of F(ab’)2 − ADCC killing measured in the presence of F(ab′)2.

### Proximity extension assay on plasma samples

Protein quantification was executed employing the Olink^®^ Target 48 Cytokine panel* (Olink Proteomics AB, Uppsala, Sweden) in accordance with the manufacturer’s protocols. This method leverages the Proximity Extension Assay (PEA) technology, as extensively detailed by *Assarsson* et al. (68). This specific PEA methodology enables the concurrent assessment of 45 distinct analytes. Briefly, pairs of oligonucleotide-labeled antibody probes, each tailored to selectively bind to their designated protein targets were used. A mixture of 3 ul probe pairs was incubated with 1 ul of plasma. Probes that encountered their cognate proteins were then in close spatial proximity and their respective oligonucleotides could engage in pair-wise hybridization. A DNA polymerase was used to amplify the polymerized DNA, and to create distinct PCR target sequences. Subsequently we detected and quantified these newly formed DNA sequences through utilization of a microfluidic real-time PCR platform, specifically the Biomark HD system by Fluidigm (Olink Signature Q100 instrument). Validation to uphold data integrity was conducted with the Olink NPX Signature software specifically designed for the Olink^®^ analysis: the application was used to import data from the Olink Signature Q100 instrument and process the data. Data normalization procedures were executed employing an internal extension control and calibrators, thereby effectively mitigating any inherent intra-run variability. The ultimate assay output was reported in picograms per milliliter (pg/ml), predicated upon a robust 4-parameter logistic (4-Pl) fit model, thereby ensuring precise absolute quantification. Comprehensive insights into the assay’s validation parameters, encompassing limits of detection, intra- and inter-assay precision data, and related metrics are available at www.olink.com.

## Data Availability

The original contributions presented in the study are included in the article/**Supplementary Material**. Further inquiries can be directed to the corresponding authors.

## Supplementary material

Supplementary Figure 1 Evaluation of memory-like NK cell responses in mucosa. **(A-D)** Evaluation of **(A)** PMA/Ionomycin -induced IFN-γ^+^ memory-like NK cells over the course of vaccination in the female macaques, and **(B)** PMA/Ionomycin -induced CD107^+^ NK cells, **(C)** PMA/Ionomycin -induced TNF-α^+^ NK cells, and **(D)** PMA/Ionomycin -induced IFN-γ^+^ memory-like NK cells over the course of vaccination of the male macaques. **(E)** Comparison of ADCC activity between naïve animals and vaccinated animals. **(F)** Correlation of memory-like NK cells with V2-specific ADCC in the male macaques. Data shown in **(A-E)** were analyzed with Wilcoxon signed-rank test or Mann-Whitney test. Data shown in **(F)** were analyzed with the Spearman correlation test. Horizontal and vertical bars denote mean and SD, respectively. Violin plot vertical bars denote median and quartiles. Here, black, red and blue symbols represent ΔV1 SIV vaccinated female macaques, WT HIV vaccinated male macaques and ΔV1 HIV vaccinated male macaques, respectively.

Supplementary Figure 2 Evaluation of antigen- reactive ILC responses in mucosa. **(A-H)** Evaluation of **(A)** ILC1, **(B)** ILC2, **(C)** ILC3, **(D)** PMA/Ionomycin-induced IFN-γ^+^ ILC1, **(E)** PMA/Ionomycin-induced IL-13^+^ ILC2, **(F)** PMA/Ionomycin-induced IL-17^+^ ILC3, **(G)** PMA/Ionomycin-induced IFN-γ^+^ ILC1 and **(H)** PMA/Ionomycin-induced CD107^+^ ILC2 over the course of the studies. **(I-N)** Correlation of **(I, J)** PMA/Ionomycin-induced IL-13^+^ ILC2, **(K)** PMA/Ionomycin-induced IFN-γ^+^ ILC1, **(L)** ILC1, and **(M, N)** env-reactive IL-17^+^ ILC3 with VL. Data shown in **(A-H)** were analyzed with Wilcoxon signed-rank test or Mann-Whitney test. Data shown in **(I-N)** were analyzed with the Spearman correlation test. Horizontal and vertical bars denote mean and SD. Violin plot vertical bars denote median and quartiles. Here, black, red and blue symbols represent ΔV1 SIV vaccinated female macaques, WT HIV vaccinated male macaques and ΔV1 HIV vaccinated male macaques, respectively.

Supplementary Figure 3 Evaluation of T cell responses in blood and their association with ILCs. **(A, B)** Evaluation of **(A)** CCR5^-^α4β7^+^ Th1 cells, **(B)** CCR5^-^α4β7^+^ Th2 cells over the course of the studies. **(C-E)** Correlation of **(C)** env-reactive IL-13^+^ ILC2 with vaccine induced CCR5^-^α4β7^+^ Th1 cells, **(D)** PMA/Ionomycin-induced IL-17^+^ ILC3 with vaccine induced CCR5^-^α4β7^+^ Th2 cells, and **(E)** env-reactive IL-17^+^ ILC3 vaccine induced CCR5^-^α4β7^+^ Th1 cells**. (F)** Evaluation of CCR5^+^α4β7^+^ Th1 cells over the course of the studies. **(G)** Correlation of env-reactive IL-17^+^ ILC3 with vaccine induced CCR5^+^α4β7^+^ Th1 cells. Data shown in **(A, B, F)** were analyzed with Wilcoxon signed-rank test. Data shown in **(C-E, G)** were analyzed with the Spearman correlation test. Horizontal and vertical bars denote mean and SD. Here, black symbols represent ΔV1 SIV vaccinated female macaques.

Supplementary Figure 4 Evaluation of NK/ILC responses in mucosa. **(A-E)** Evaluation of **(A)** NKG2A^+^ NK cells, **(B)** env-reactive IFN-γ^+^ NKG2A^+^ NK cells, **(C, D)** PMA/Ionomycin-induced IFN-γ^+^ NKG2A^+^ NK cells, and **(E)** NKp44^+^ ILCs over the course of the study. **(F)** Correlation of NKp44^+^ ILCs with vaccine induced Th17 cells. **(G, H)** Comparison of PMA/Ionomycin-induced IL-17^+^ NKp44^+^ ILCs over the course of the studies. **(I, J)** Correlation of PMA/Ionomycin-induced IL-17^+^ NKp44^+^ ILCs with number of challenges in the **(I)** female and **(J)** male macaques. **(K, L)** Correlation of PMA/Ionomycin-induced IL-17^+^ NKp44^+^ ILCs with **(K)** female V2-specific ADCC and **(L)** vaccine induced CCR5^+^α4β7^+^ Th1 cells in the female macaques. Data shown in **(A-E, G, H)** were analyzed with Wilcoxon signed-rank test or Mann-Whitney test. Data shown in **(F, I-L)** were analyzed by the Spearman correlation test. Horizontal and vertical bars denote mean and SD. Violin plot vertical bars denote median and quartiles. Here, gray, red and blue symbols represent ΔV1 SIV vaccinated female macaques, WT HIV vaccinated male macaques and ΔV1 HIV vaccinated male macaques, respectively.

Supplementary Figure 5 Evaluation of NKG2A^-^NKP44^-^ ILC responses in mucosa. **(A-E)** Evaluation of **(A)** NKG2A^-^NKp44^-^ ILCs, **(B)** env-reactive IFN-γ^+^ NKG2A^-^NKp44^-^ ILCs, **(C, D)** PMA/Ionomycin-induced IFN-γ^+^ NKG2A^-^NKp44^-^ ILCs, and **(E)** PMA/Ionomycin-induced CD107^+^ NKG2A^-^NKp44^-^ ILCs over the course of the studies. **(F, G)** Correlation of env-reactive IFN-γ^+^ NKG2A^-^NKp44^-^ ILCs with **(F)** CCR5^-^α4β7^+^ Th2 cells and **(G)** CCR5^-^α4β7^-^ Th2 cells. **(H)** Correlation of PMA/Ionomycin-induced IFN-γ^+^ NKG2A^-^NKp44^-^ ILCs with vaccine induced Th2 cells. **(I, J)** Correlation of PMA/Ionomycin-induced IFN-γ^+^ NKG2A^-^NKp44^-^ ILCs with number of challenges in the female and male macaques, respectively. **(K)** Correlation of PMA/Ionomycin-induced CD107^+^ NKG2A^-^NKp44^-^ ILCs with number of challenges in the male macaques. Data shown in **(A-E)** were analyzed with Wilcoxon signed-rank test or Mann-Whitney test. Data shown in **(F-K)** were analyzed by the Spearman correlation test. Horizontal and vertical bars denote mean and SD. Violin plot vertical bars denote median and quartiles. Here, gray, red and blue symbols represent ΔV1 SIV vaccinated female macaques, WT HIV vaccinated male macaques and ΔV1 HIV vaccinated male macaques, respectively.

Supplementary Figure 6 Evaluation of systemic NK/ILC responses in female macaques. **(A-G)** Evaluation of **(A)** NKG2A^+^ NK cells, **(B)** env-reactive GranB^+^ NK cells, **(C)** env-reactive IFN-γ^+^ NK cells, **(D)** PMA/Ionomycin-induced GranB^+^ memory-like NK cells, **(E)** PMA/Ionomycin-induced IFN-γ^+^ memory-like NK cells, **(F)** PMA/Ionomycin-induced GranB^+^ NK cells, and **(G)** PMA/Ionomycin-induced IFN-γ^+^ NK cells over the course of vaccination of female macaques. **(H)** Correlation of systemic PMA/Ionomycin-induced GranB^+^ memory-like NK cells with number of challenges. **(I)** Evaluation of ILC2 over the course of vaccination of female macaques. Violin plot data shown in **(A-G, I)** were analyzed with Wilcoxon signed-rank test. Data shown in **(H)** were analyzed by the Spearman correlation test. Violin plot vertical bars denote median and quartiles. Here, black/gray symbols represent ΔV1 SIV vaccinated female macaques.

Supplementary Figure 7 Association of cytokines/chemokines with mucosal/systemic immune responses. **(A-C)** Correlation of systemic PMA/Ionomycin-induced GranB^+^ memory-like NK cell frequency with **(A)** IL-33, **(B)** CCL-13, and **(C)** MMP-1. **(D, E)** Correlation of mucosal PMA/Ionomycin-induced IL-17^+^ NKp44^+^ ILCs with D) TNFSF-12, and **(E)** IL-15. **(F, G)** Correlation of TNF with **(F)** mucosal PMA/Ionomycin-induced CD107^+^ NKG2A^-^NKP44^-^ ILCs and **(G)** mucosal PMA/Ionomycin-induced IFN-γ^+^NKG2A^-^NKP44^-^ ILCs. Data shown in **(A-G)** were analyzed by the Spearman correlation test. Here, black, red and blue symbols represent ΔV1 SIV vaccinated female macaques, WT HIV vaccinated male macaques and ΔV1 HIV vaccinated male macaques, respectively.
