# Supplementary figures and images for: Vaccine induced mucosal and systemic memory NK/ILCs elicit decreased risk of SIV/SHIV acquisition
Source: Front Immunol. 2024 Sep 5;15:1441793. doi: 10.3389/fimmu.2024.1441793 (PMC11410642; doi:10.3389/fimmu.2024.1441793)

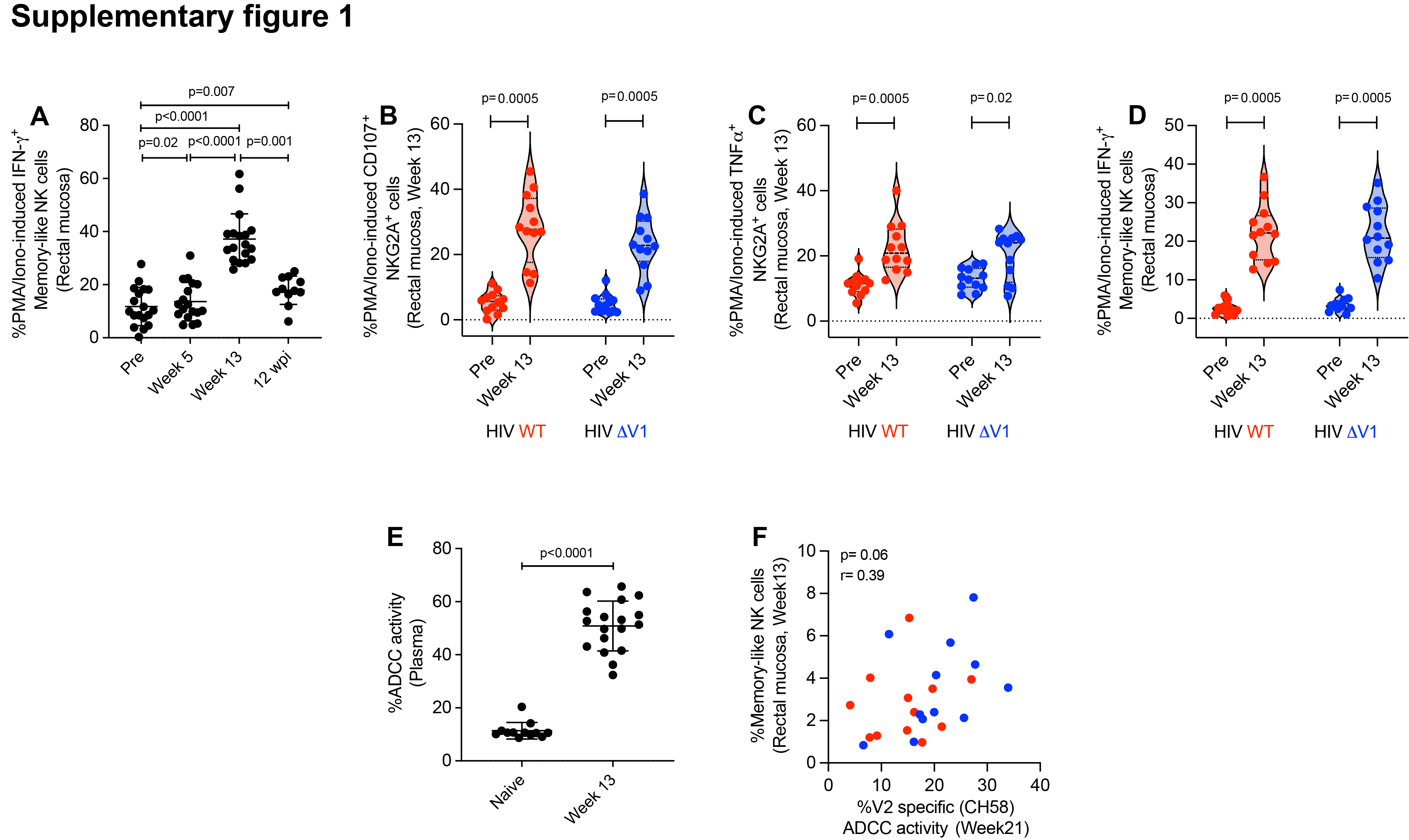

Supplement: Supplementary Figure 1 — Evaluation of memory-like NK cell responses in mucosa. (A-D) Evaluation of (A) PMA/Ionomycin -induced IFN-γ+ memory-like NK cells over the course of vaccination in the female macaques, and (B) PMA/Ionomycin -induced CD107+ NK cells, (C) PMA/Ionomycin -induced TNF-α+ NK cells, and (D) PMA/Ionomycin -induced IFN-γ+ memory-like NK cells over the course of vaccination of the male macaques. (E) Comparison of ADCC activity between naïve animals and vaccinated animals. (F) Correlation of memory-like NK cells with V2-specific ADCC in the male macaques. Data shown in (A-E) were analyzed with Wilcoxon signed-rank test or Mann-Whitney test. Data shown in (F) were analyzed with the Spearman correlation test. Horizontal and vertical bars denote mean and SD, respectively. Violin plot vertical bars denote median and quartiles. Here, black, red and blue symbols represent ΔV1 SIV vaccinated female macaques, WT HIV vaccinated male macaques and ΔV1 HIV vaccinated male macaques, respectively. [file Image1.jpeg]

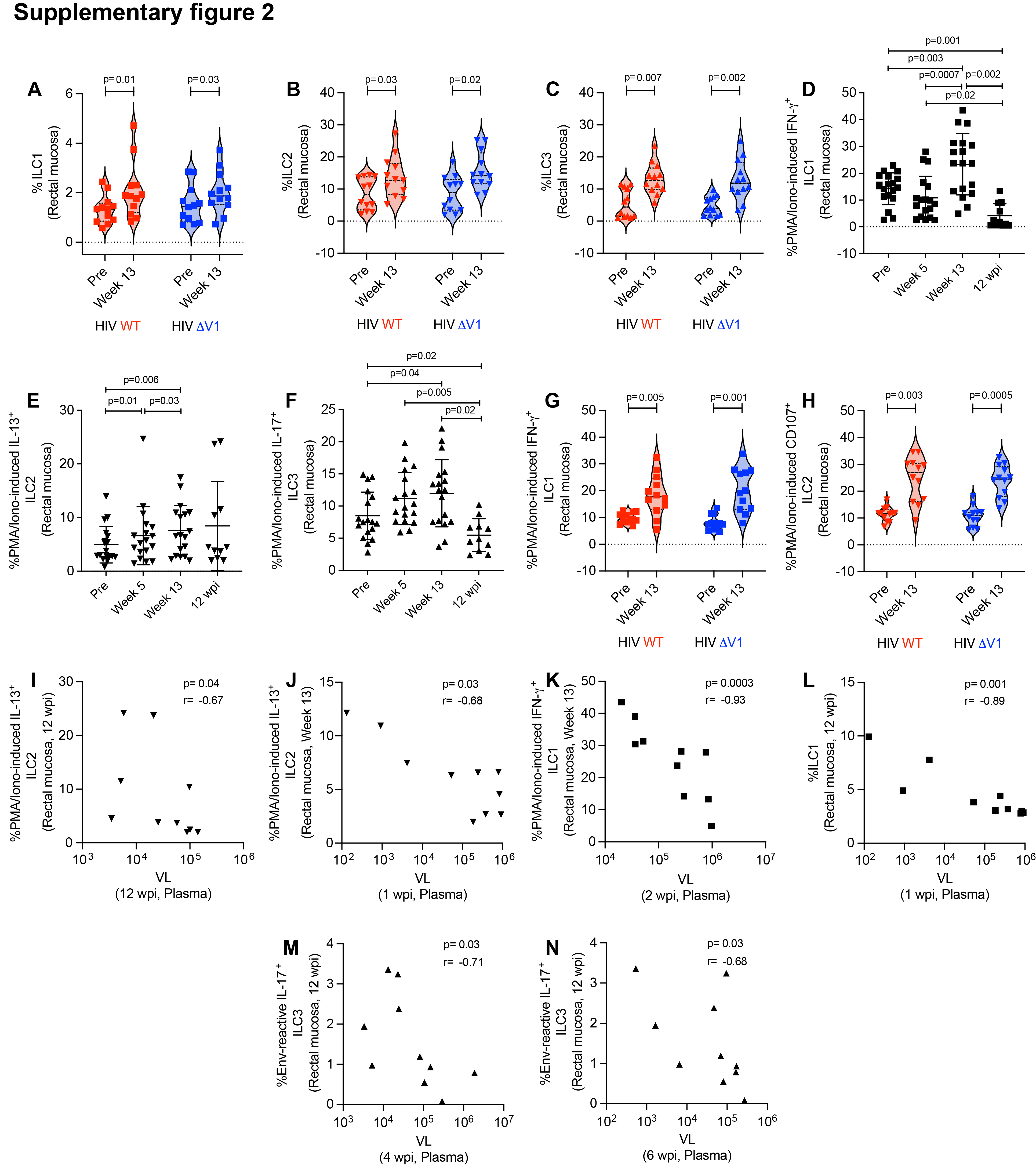

Supplement: Supplementary Figure 2 — Evaluation of antigen- reactive ILC responses in mucosa. (A-H) Evaluation of (A) ILC1, (B) ILC2, (C) ILC3, (D) PMA/Ionomycin-induced IFN-γ+ ILC1, (E) PMA/Ionomycin-induced IL-13+ ILC2, (F) PMA/Ionomycin-induced IL-17+ ILC3, (G) PMA/Ionomycin-induced IFN-γ+ ILC1 and (H) PMA/Ionomycin-induced CD107+ ILC2 over the course of the studies. (I-N) Correlation of (I, J) PMA/Ionomycin-induced IL-13+ ILC2, (K) PMA/Ionomycin-induced IFN-γ+ ILC1, (L) ILC1, and (M, N) env-reactive IL-17+ ILC3 with VL. Data shown in (A-H) were analyzed with Wilcoxon signed-rank test or Mann-Whitney test. Data shown in (I-N) were analyzed with the Spearman correlation test. Horizontal and vertical bars denote mean and SD. Violin plot vertical bars denote median and quartiles. Here, black, red and blue symbols represent ΔV1 SIV vaccinated female macaques, WT HIV vaccinated male macaques and ΔV1 HIV vaccinated male macaques, respectively. [file Image2.jpeg]

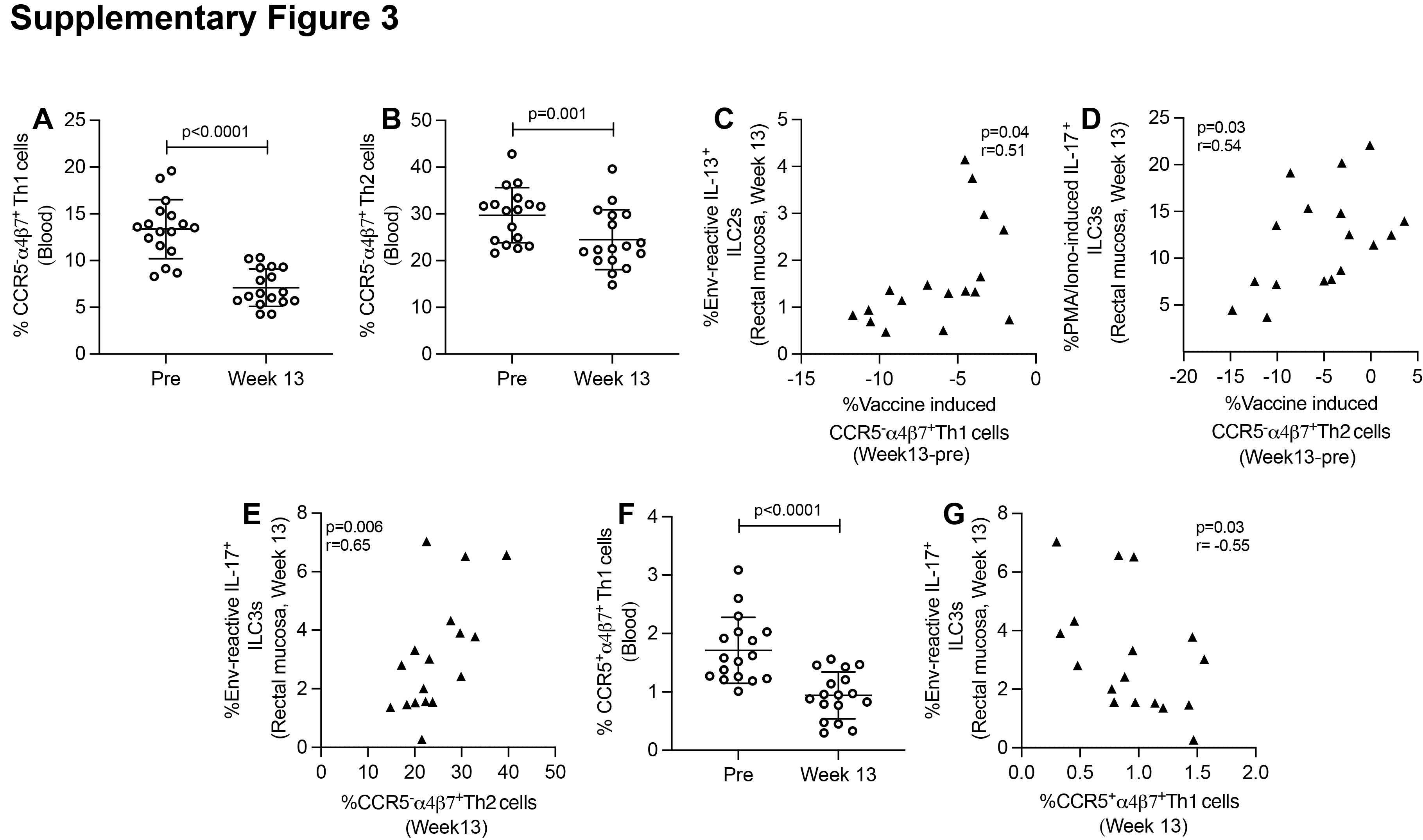

Supplement: Supplementary Figure 3 — Evaluation of T cell responses in blood and their association with ILCs. (A, B) Evaluation of (A) CCR5-α4β7+ Th1 cells, (B) CCR5-α4β7+ Th2 cells over the course of the studies. (C-E) Correlation of (C) env-reactive IL-13+ ILC2 with vaccine induced CCR5-α4β7+ Th1 cells, (D) PMA/Ionomycin-induced IL-17+ ILC3 with vaccine induced CCR5-α4β7+ Th2 cells, and (E) env-reactive IL-17+ ILC3 vaccine induced CCR5-α4β7+ Th1 cells. (F) Evaluation of CCR5+α4β7+ Th1 cells over the course of the studies. (G) Correlation of env-reactive IL-17+ ILC3 with vaccine induced CCR5+α4β7+ Th1 cells. Data shown in (A, B, F) were analyzed with Wilcoxon signed-rank test. Data shown in (C-E, G) were analyzed with the Spearman correlation test. Horizontal and vertical bars denote mean and SD. Here, black symbols represent ΔV1 SIV vaccinated female macaques. [file Image3.jpeg]

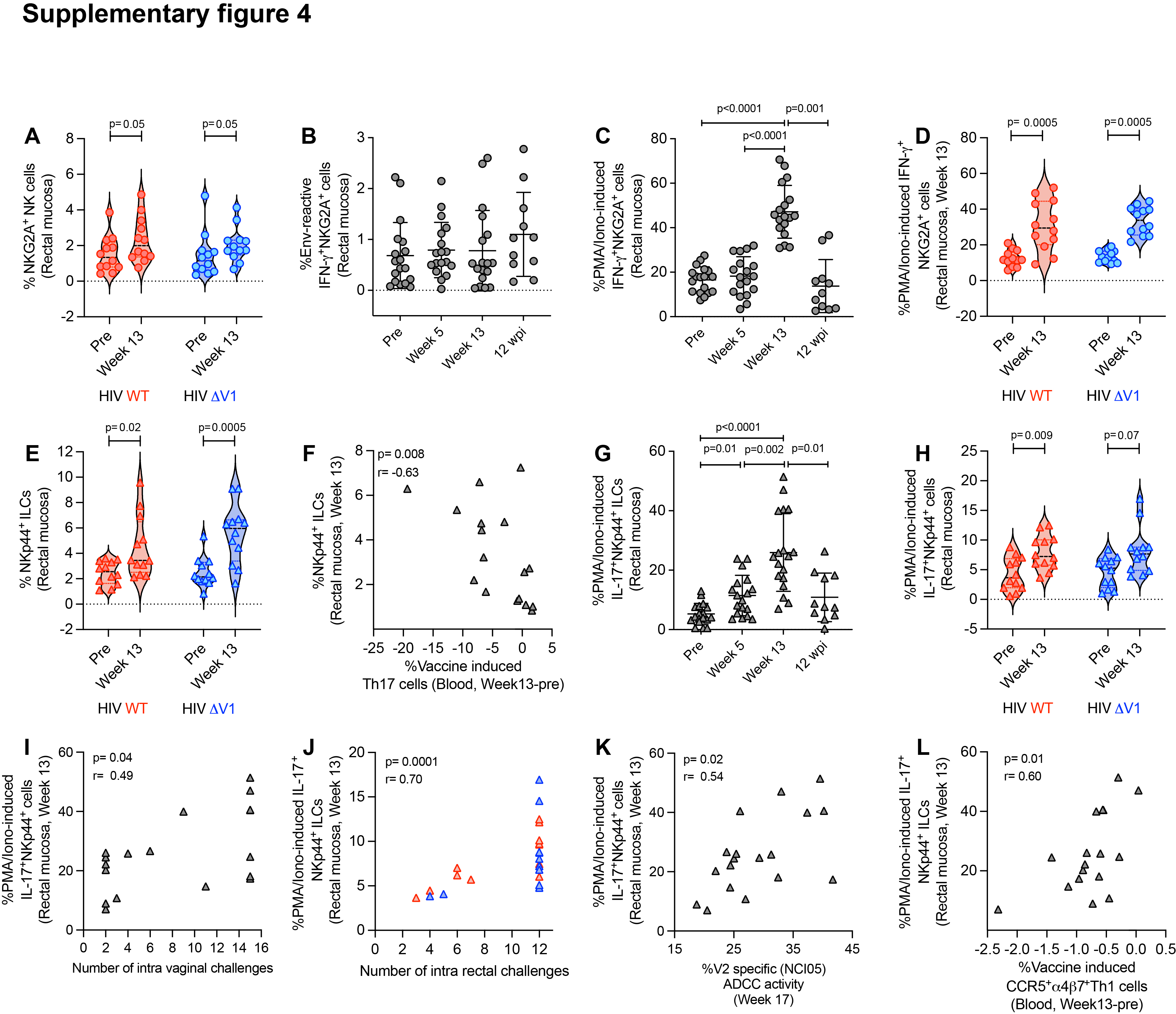

Supplement: Supplementary Figure 4 — Evaluation of NK/ILC responses in mucosa. (A-E) Evaluation of (A) NKG2A+ NK cells, (B) env-reactive IFN-γ+ NKG2A+ NK cells, (C, D) PMA/Ionomycin-induced IFN-γ+ NKG2A+ NK cells, and (E) NKp44+ ILCs over the course of the study. (F) Correlation of NKp44+ ILCs with vaccine induced Th17 cells. (G, H) Comparison of PMA/Ionomycin-induced IL-17+ NKp44+ ILCs over the course of the studies. (I, J) Correlation of PMA/Ionomycin-induced IL-17+ NKp44+ ILCs with number of challenges in the (I) female and (J) male macaques. (K, L) Correlation of PMA/Ionomycin-induced IL-17+ NKp44+ ILCs with (K) female V2-specific ADCC and (L) vaccine induced CCR5+α4β7+ Th1 cells in the female macaques. Data shown in (A-E, G, H) were analyzed with Wilcoxon signed-rank test or Mann-Whitney test. Data shown in (F, I-L) were analyzed by the Spearman correlation test. Horizontal and vertical bars denote mean and SD. Violin plot vertical bars denote median and quartiles. Here, gray, red and blue symbols represent ΔV1 SIV vaccinated female macaques, WT HIV vaccinated male macaques and ΔV1 HIV vaccinated male macaques, respectively. [file Image4.jpeg]

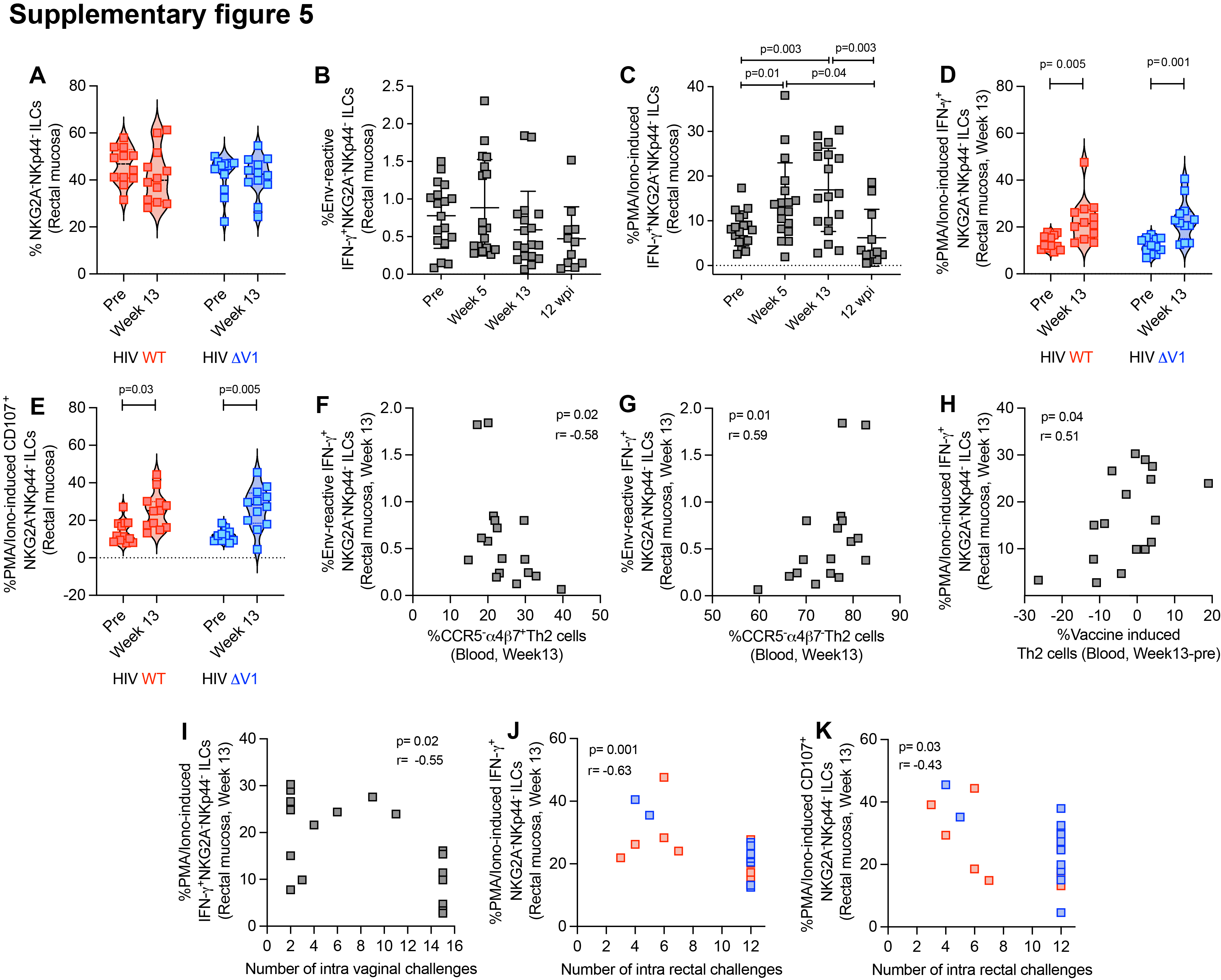

Supplement: Supplementary Figure 5 — Evaluation of NKG2A-NKP44- ILC responses in mucosa. (A-E) Evaluation of (A) NKG2A-NKp44- ILCs, (B) env-reactive IFN-γ+ NKG2A-NKp44- ILCs, (C, D) PMA/Ionomycin-induced IFN-γ+ NKG2A-NKp44- ILCs, and (E) PMA/Ionomycin-induced CD107+ NKG2A-NKp44- ILCs over the course of the studies. (F, G) Correlation of env-reactive IFN-γ+ NKG2A-NKp44- ILCs with (F) CCR5-α4β7+ Th2 cells and (G) CCR5-α4β7- Th2 cells. (H) Correlation of PMA/Ionomycin-induced IFN-γ+ NKG2A-NKp44- ILCs with vaccine induced Th2 cells. (I, J) Correlation of PMA/Ionomycin-induced IFN-γ+ NKG2A-NKp44- ILCs with number of challenges in the female and male macaques, respectively. (K) Correlation of PMA/Ionomycin-induced CD107+ NKG2A-NKp44- ILCs with number of challenges in the male macaques. Data shown in (A-E) were analyzed with Wilcoxon signed-rank test or Mann-Whitney test. Data shown in (F-K) were analyzed by the Spearman correlation test. Horizontal and vertical bars denote mean and SD. Violin plot vertical bars denote median and quartiles. Here, gray, red and blue symbols represent ΔV1 SIV vaccinated female macaques, WT HIV vaccinated male macaques and ΔV1 HIV vaccinated male macaques, respectively. [file Image5.jpeg]

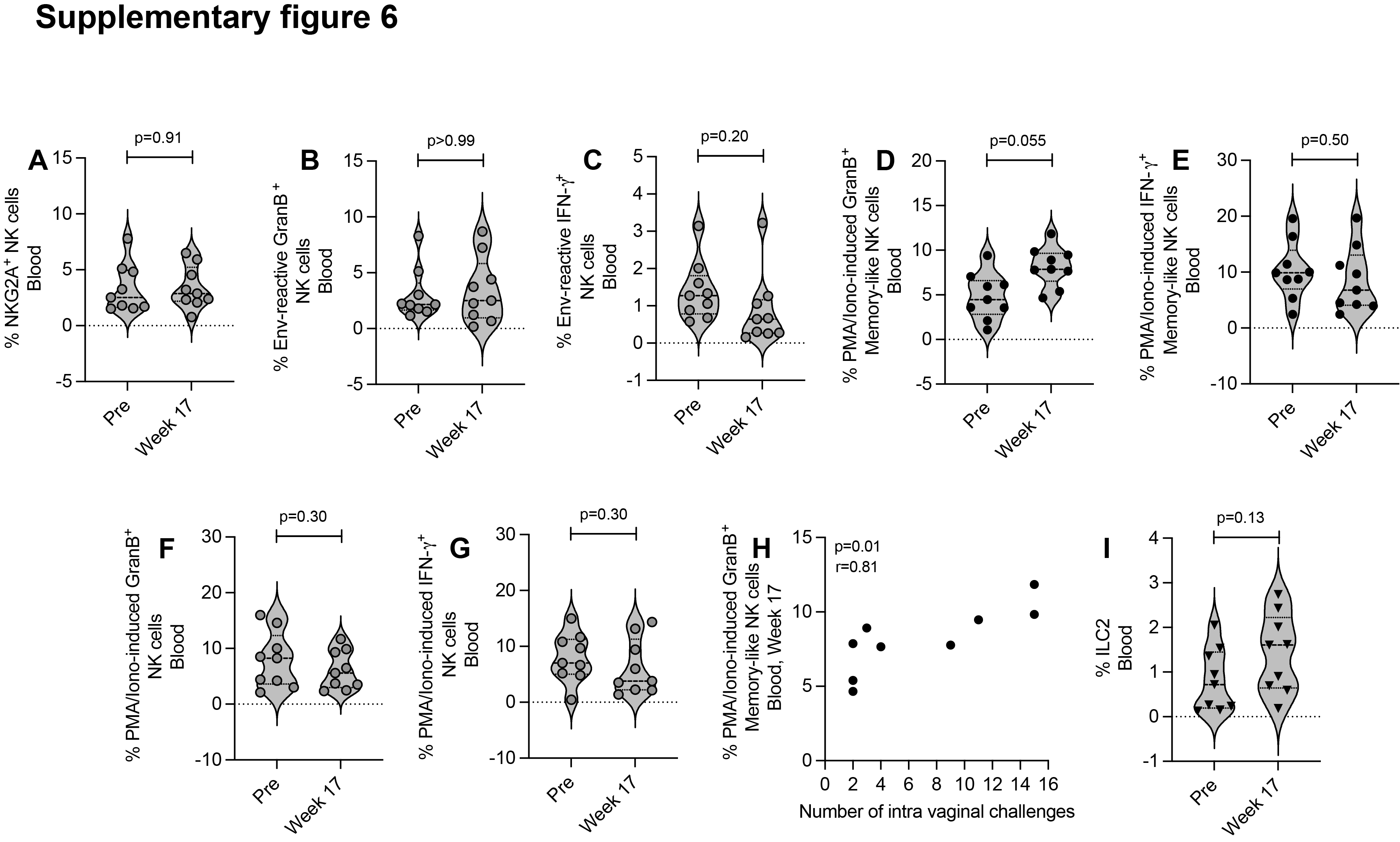

Supplement: Supplementary Figure 6 — Evaluation of systemic NK/ILC responses in female macaques. (A-G) Evaluation of (A) NKG2A+ NK cells, (B) env-reactive GranB+ NK cells, (C) env-reactive IFN-γ+ NK cells, (D) PMA/Ionomycin-induced GranB+ memory-like NK cells, (E) PMA/Ionomycin-induced IFN-γ+ memory-like NK cells, (F) PMA/Ionomycin-induced GranB+ NK cells, and (G) PMA/Ionomycin-induced IFN-γ+ NK cells over the course of vaccination of female macaques. (H) Correlation of systemic PMA/Ionomycin-induced GranB+ memory-like NK cells with number of challenges. (I) Evaluation of ILC2 over the course of vaccination of female macaques. Violin plot data shown in (A-G, I) were analyzed with Wilcoxon signed-rank test. Data shown in (H) were analyzed by the Spearman correlation test. Violin plot vertical bars denote median and quartiles. Here, black/gray symbols represent ΔV1 SIV vaccinated female macaques. [file Image6.jpeg]

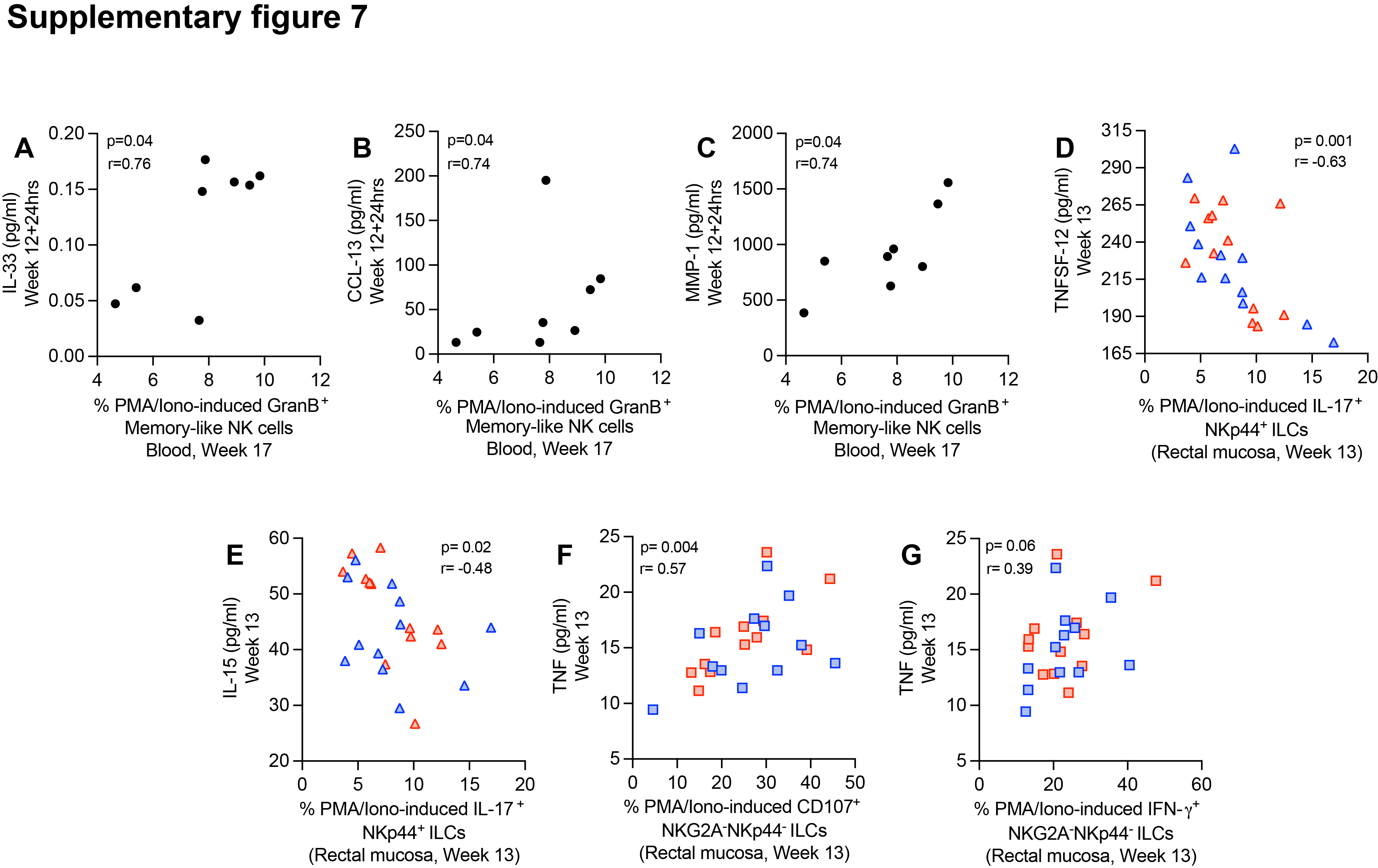

Supplement: Supplementary Figure 7 — Association of cytokines/chemokines with mucosal/systemic immune responses. (A-C) Correlation of systemic PMA/Ionomycin-induced GranB+ memory-like NK cell frequency with (A) IL-33, (B) CCL-13, and (C) MMP-1. (D, E) Correlation of mucosal PMA/Ionomycin-induced IL-17+ NKp44+ ILCs with D) TNFSF-12, and (E) IL-15. (F, G) Correlation of TNF with (F) mucosal PMA/Ionomycin-induced CD107+ NKG2A-NKP44- ILCs and (G) mucosal PMA/Ionomycin-induced IFN-γ+NKG2A-NKP44- ILCs. Data shown in (A-G) were analyzed by the Spearman correlation test. Here, black, red and blue symbols represent ΔV1 SIV vaccinated female macaques, WT HIV vaccinated male macaques and ΔV1 HIV vaccinated male macaques, respectively. [file Image7.jpeg]
